# Blockade of sustained tumor necrosis factor in a transgenic model of progressive autoimmune encephalomyelitis limits oligodendrocyte apoptosis and promotes oligodendrocyte maturation

**DOI:** 10.1186/s12974-018-1164-y

**Published:** 2018-04-24

**Authors:** Alice Valentin-Torres, Carine Savarin, Joslyn Barnett, Cornelia C. Bergmann

**Affiliations:** 1 Department of Neurosciences NC-30, Lerner Research Institute, The Cleveland Clinic, 9500 Euclid Ave., Cleveland, OH 44195 USA; 2 Department of Regenerative Medicine, Athersys, Inc., 3201 Carnegie Ave., Cleveland, OH 44115-2634 USA; 3 Department of Molecular, Cellular, and Developmental Biology, Yale University, New Haven, CT 06520 USA

**Keywords:** Progressive multiple sclerosis, Experimental autoimmune encephalomyelitis, Tumor necrosis factor, Astrocytes, Oligodendrocytes, Endothelin-1

## Abstract

**Background:**

Tumor necrosis factor (TNF) is associated with several neurodegenerative disorders including multiple sclerosis (MS). Although TNF-targeted therapies have been largely unsuccessful in MS, recent preclinical data suggests selective soluble TNF inhibition can promote remyelination. This has renewed interest in regulation of TNF signaling in demyelinating disease, especially given the limited treatment options for progressive MS. Using a mouse model of progressive MS, this study evaluates the effects of sustained TNF on oligodendrocyte (OLG) apoptosis and OLG precursor cell (OPC) differentiation.

**Methods:**

Induction of experimental autoimmune encephalomyelitis (EAE) in transgenic mice expressing a dominant-negative interferon-γ receptor under the human glial fibrillary acidic protein promoter (GFAPγR1Δ) causes severe non-remitting disease associated with sustained TNF. Therapeutic effects in GFAPγR1Δ mice treated with anti-TNF compared to control antibody during acute EAE were evaluated by assessing demyelinating lesion size, remyelination, OLG apoptosis, and OPC differentiation.

**Results:**

More severe and enlarged demyelinating lesions in GFAPγR1Δ compared to wild-type (WT) mice were associated with increased OLG apoptosis and reduced differentiated CC1^+^Olig2^+^ OLG within lesions, as well as impaired upregulation of TNF receptor-2, suggesting impaired OPC differentiation. TNF blockade during acute EAE in GFAPγR1Δ both limited OLG apoptosis and enhanced OPC differentiation consistent with reduced lesion size and clinical recovery. TNF neutralization further limited increasing endothelin-1 (ET-1) expression in astrocytes and myeloid cells noted in lesions during disease progression in GFAPγR1Δ mice, supporting inhibitory effects of ET-1 on OPC maturation.

**Conclusion:**

Our data implicate that IFNγ signaling to astrocytes is essential to limit a detrimental positive feedback loop of TNF and ET-1 production, which increases OLG apoptosis and impairs OPC differentiation. Interference of this cycle by TNF blockade promotes repair independent of TNFR2 and supports selective TNF targeting to mitigate progressive forms of MS.

**Electronic supplementary material:**

The online version of this article (10.1186/s12974-018-1164-y) contains supplementary material, which is available to authorized users.

## Background

Multiple sclerosis (MS) is a chronic inflammatory disease associated with focal demyelinating lesions in white and gray matter of the brain (BR) and spinal cord (SC) [1–7]. Active lesions characteristic of relapsing remitting MS (RR-MS) contain leukocyte infiltrates and are associated with blood-brain barrier (BBB) leakage, loss of mature oligodendrocytes (OLG), and partial axonal damage, which is balanced by endogenous repair. Remyelination by OLG progenitors cells (OPC) requires clearance of myelin debris, migration of OPC to areas of damaged myelin, and their proliferation and differentiation to newly myelinating cells [8–13]. Irrespective of initially effective treatment options prolonging the onset of severe neurological cognitive and motor impairments in patients with RR-MS, therapeutic interventions fail in patients with primary and secondary progressive (P)-MS. This disease state is characterized by sparse inflammation, lack of remyelination, mitochondrial dysfunction, axonal loss, and CNS atrophy [1–7, 14]. Progress in understanding mechanisms underlying irreversible disease progression has been hampered by the lack of animal models exhibiting the pathology and progressive disability observed in P-MS patients [15, 16]. Further, while much attention has focused on detrimental functions of pro-inflammatory factors, monocytes, and microglia, less is known about the transition to repair functions by glia and mechanisms shifting the balance to irreversible damage. For example, astrocytes, interferon γ (IFNγ), and tumor necrosis factor (TNF) individually are all known to promote inflammation but also mediate neuroprotective functions in demyelinating disease [17–21].

Astrocytes play a vital role in maintaining CNS homeostasis and display a wide range of functions in response to injury and subsequent disease processes, including demyelination. These range from altering morphology and biochemical metabolism, inducing pro- and anti-inflammatory responses, as well as cytokines guiding peripheral immune cells or resident OPC for repair, directly enhancing or inhibiting remyelination, and forming glial scars [19]. In MS, activated astrocytes can also take on several morphological states depending on lesion activity [4, 9, 21, 22], While it is thus apparent that astrocytes display highly dynamic functions guided by their integration of stimuli in the local environment at various stages of disease [23–25], the extent of their transient or permanent adaptation and function once injury or inflammation subsides requires better characterization.

We have recently reported that transgenic mice, in which astrocytes are unable to respond to IFNγ by expression of a dominant negative IFNγR receptor capable of IFNγ binding, but not signaling (GFAPγR1∆), develop a chronic non-resolving form of EAE [26]. This progressive disease recapitulates some hallmarks of P-MS including increasing morbidity associated with extensive spinal cord white matter demyelination, axonal damage, and astrogliosis in white and gray matter. The inability of GFAPγR1Δ mice to resolve acute EAE was associated with elevated inflammation, including IL-6, and sustained TNF production specifically by macrophages [26–28]. Disease-enhancing activity of TNF is supported by TNF in active MS lesions [29] as well as elevated TNF in serum and cerebral spinal fluid [30]. In rodents, TNF in the CNS correlates with EAE disease [31] and transgenic TNF expression within the CNS leads to demyelinating disease [32–34]. TNF can not only directly induce OLG death [35–42], but also inflict excitotoxic damage to OLG and neurons by modulating the release of glutamate from astrocytes [35, 39], thereby impairing OPC differentiation [40]. Consistent with a detrimental role of sustained elevated TNF in GFAPγR1Δ mice undergoing EAE, TNF neutralization initiated at the peak of acute disease ameliorated disease and demyelination, limited CNS cellular infiltrates indiscriminately without affecting peripheral T cell responses, and limited BBB breakdown [28]. Similar anti-TNF treatment in WT mice did not accelerate EAE disease remission [28]. Furthermore, in MS patients, anti-TNF therapy failed to mitigate RR-MS and even worsened disease [43]. These contrasting results and historic setbacks in targeting TNF in MS patients have since been attributed to distinct TNF activities mediated by interactions of soluble (s)TNF and its transmembrane (tm)TNF precursor with their preferential TNFR1 and TNFR2 receptors: sTNF with higher affinity for TNFR1 mediates apoptosis and chronic inflammation [17]. Conversely, tmTNF with higher affinity for TNFR2 activates genes important for cell survival, resolution of inflammation, and OPC differentiation [17, 44–49]. By promoting remyelination [17, 44–49] as well as T_reg_ differentiation and survival [50, 51], TNF/TNFR2 signaling thus acts at multiple levels to enhance protection and repair. Blockade or genetic manipulation of distinct TNF activities in EAE has substantiated the dual pro- and anti-inflammatory activities of TNF in regulating disease activity [48, 52, 53].

The difficulties in targeting TNF as a therapeutic strategy to limit demyelination and favor remyelination thus clearly depend on the interplay of both TNF forms with their respective receptors on distinct cell types. The effectiveness of anti-TNF mAb therapy on EAE progression in our GFAPγR1Δ mouse model without evidence of detrimental effects supported the notion that the TNFR2 pathway may be overwhelmed once a pathological threshold is reached. Herein, we examined how TNF blockade during acute EAE in GFAPγR1Δ mice affects both de- and re-myelination. Distinct from a reduction in demyelinating lesion size during EAE disease remission in WT mice, lesion size increased during progressive EAE in GFAPγR1Δ mice, suggesting ongoing demyelination overrides any efforts of remyelination. Anti-TNF treatment significantly reduced lesion size coincident with reduced astrocyte and macrophage/microglia reactivity within demyelinating lesions. Moreover, anti-TNF mAb not only limited OLG apoptosis, but also promoted OPC differentiation within lesioned areas in GFAPγR1Δ mice. The implication that sTNF/TNFR1 mediated OLG death and inhibition of OPC differentiation dominates over TNFR2-mediated repair during disease progression in GFAPγR1Δ mice was supported by reduced TNFR2 relative to TNFR1 mRNA expression compared to remitting EAE in WT mice. Importantly, TNF blockade further limited expression of the vasoconstrictive peptide endothelin 1 (ET-1) [54], which was vastly upregulated in lesions during progressive EAE in GFAPγR1Δ mice. Taking into account the inhibitory effects of ET-1 on OPC maturation [55], our data demonstrate that TNF blockade limits progressive demyelination by reducing OLG death and increasing OPC differentiation, thereby promoting repair during progressive EAE. These dual effects support TNF as a potential target for therapeutic approaches in progressive forms of MS.

## Methods

### Mice

Homozygous H-2^b^ GFAP/IFNγR1ΔIC (GFAPγR1Δ) transgenic mice expressing a dominant negative IFNγ receptor alpha chain under the human glial fibrillary acidic protein (GFAP) promoter were previously described and bred locally [26, 56]. C57BL/6 (H-2^b^) WT mice were purchased from The Jackson Laboratories (Bar Harbor, ME).

### EAE and anti-TNF treatment

EAE was induced in 7–8-week-old mice as previously described [26–28]. Briefly, mice were immunized subcutaneously in the right flanks with 200 μl of myelin oligodendrocyte glycoprotein (MOG)^35–55^ peptide (Biosynthesis, Lewisville, TX) at 3 mg/ml in an emulsion of PBS and an equal volume of incomplete Freund’s adjuvant (IFA; Sigma-Aldrich, St. Louis, MO) supplemented with 5 mg/ml of *Mycobacterium tuberculosis* strain H37Ra (Difco, Detroit, MI). At day 0 and day 2 post-immunization (p.i.), mice also received 200 ng of pertussis toxin intraperitoneally (i.p.). At d7 p.i., a second dose of 200 μl MOG was administered into the left flank. Clinical symptoms were monitored daily using the following scale: 0 = no signs of disease, 1 = flaccid tail, 2 = flaccid tail and partial hind limb paralysis, 3 = complete hind limb paralysis, 4 = moribund state, and 5 = dead.

Mice received 0.5 mg of either neutralizing rat anti-murine TNF (clone MD6-XT3.11, BioXCell, West Lebanon, NH) or control rat IgG1 anti-β-galactosidase (clone GL113, gift from Dr. Robert Coffman, DNAX Corp, Palo Alto, CA) monoclonal antibody (mAb) i.p. starting at the peak of acute disease (d19 p.i.) followed by injections every 2 days for a total of four doses as described [28]. The anti-TNF mAb penetrated the CNS as monitored by positive reactivity for rat Ig in brain supernatants by ELISA (data not shown).

### Immunohistochemistry and microscopy

Spinal cords from WT and GFAPγR1Δ mice perfused with ice-cold PBS during acute (d19 p.i.) and chronic (d30 p.i.) EAE were collected in OCT embedding compound (Scigen Scientific, Gardena, CA). Frozen longitudinal sections (cut at 12-μm thickness) were fixed for 20 min in 4% paraformaldehyde (PFA) followed by permeabilization with ice-cold 1% Triton x-100 (Sigma-Aldrich) for 20 min. For mouse anti-mouse APC (1:500, CC1, Millipore, Burlington, MA) and rabbit anti-mouse Olig2 (1:500, Calbiochem, Temecula, CA) staining, sections were permeabilized using sodium citrate (0.01 M, pH 6.0) antigen retrieval, subsequently blocked using 5% BSA 10% goat serum at room temperature for 1 h and then stained with primary Ab overnight at 4 °C. Immunoreactivity was visualized using fluorescently labeled secondary Abs. All primary and secondary Abs and respective dilutions as well as Neuroscience Information Framework (NIF) antibody registry number are listed in Table 1. Nuclear staining was performed using Prolong Gold with 4′,6-diamidino-2-phenylindole (DAPI) (Molecular Probes, Eugene, OR). OPCs were identified as CC1^−^ Olig2^+^, differentiated myelinating OLG as CC1^+^Olig2^+^, and more mature myelinating OLG as CC1^+^Olig2^−^ following previously described characterization [55, 57, 58].

**Table 1:** List of antibodies used for IHC analysis

	1. Antibodies							
Specificity	Iba1	GFAP	CC1	Olig2	ET-1	EDNR_B_	GFAP	CD11b
Host species*	Rb	Rb	Ms	Rb	Rb	Rb	Rt	Rt
Dilution	1:1000	1:5000	1:500	1:500	1:200	1:300	1:500	1:300
Permeabilization	1% Triton	1% Triton	0.01 M sodium citrate	0.01 M sodium citrate	1% Triton	1% Triton	1% Triton	1% Triton
Company	Wako Chemicals	Dako	Millipore	Calbiochem	Abbiotec	Abcam	Life Technologies	Invitrogen
NIF Antibody Registry	AB_839504	AB_10013382	AB_2057371	AB_570666	AB_2096229	AB_10902070	AB_86543	AB_2539241
	2. Antibodies Alexa Fluor (host-goat)							
Fluorochrome	488	488	488	594	647			
Specificity*	Ms	Rb	Rt	Rb	Rb			
NIF Antibody Registry	AB_2534069	AB_2576217	AB_2534074	AB_2556545	AB_2633282			
Dilution	1:1000							
Company	Molecular probes							

*Antibody species: Rabbit-Rb, Mouse-Ms, and Rat-Rt

Fluoromyelin red fluorescent myelin stain (1:300, Molecular Probes) was used to identify demyelinating lesions within spinal cords as directed by the manufacturer. Lesion size was determined in five to six mice per group of two to three independent experiments with at least two to three fields per spinal cord using ImageJ (NIH, Bethesda, MD). Apoptotic cells in spinal cord sections were labeled using the in Situ Cell Death Detection Kit, TMR red (TdT-mediated dUTP-X nick end labeling, TUNEL) (Roche Diagnostics, Mannheim, Germany), following the manufacturer’s instructions. All images were captured using a multiphoton microscope (Leica TCS SP5 II, Lawrenceville, GA) and analyzed using ImageJ.

The proportion of ET-1 reactivity colocalizing with either GFAP or CD11b was assessed by dividing the ET-1^+^GFAP^+^ or ET-1^+^CD11b^+^ area, respectively, by the total ET-1^+^ area. Similarly, the percentage of either GFAP or CD11b reactivity costaining for ET-1 within demyelinated areas was calculated by divided by the ET-1^+^GFAP^+^ or ET-1^+^CD11b^+^ area by the total GFAP^+^ or CD11b^+^ area, respectively. All analyses were performed using ImageJ.

### Flow cytometry

Infiltrating macrophages and microglia were enumerated by flow cytometry as previously described [28]. Briefly, at d30 p.i., brains from mice perfused with ice-cold PBS were individually homogenized in 4 ml of RPMI medium containing 25 mM HEPES, pH 7.2, using chilled Tenbroeck tissue grinders. To separate myelin debris, homogenates were resuspended in 30% Percoll (Amersham Biosciences, Piscataway, NJ), underlaid with 1 ml 70% Percoll, and isolated from the 30/70% interface following centrifugation at 800×*g* for 30 min at 4 °C. Following washing of cells, non-specific antibody binding was prevented by incubation in FACS buffer supplemented with anti-CD16/CD32 (2.4G2, BD Biosciences, San Diego, CA) and 10% of mixture of normal goat, human, mouse, and rat serum for 15 min on ice. Cells were stained with anti-CD45 (30-F11, BD Biosciences) APC and anti-CD11b (M1/70, BD Biosciences) PE mAb for 25 min on ice. Flow cytometric analysis was performed using a FACSCalibur (BD Biosciences, San Diego, CA). Samples were analyzed using FlowJo (FlowJo LLC, Ashland, OR). CD45^int^ CD11b^+^ microglia were distinguished from CD45^hi^ CD11b^+^ macrophages based on their differential CD45 expression as shown previously [28].

### Gene expression

Snap-frozen spinal cords from PBS-perfused mice were homogenized in Trizol (Invitrogen) as described [28]. RNA was extracted according to the manufacturer’s instructions, treated with DNAse I (Ambion, Austin, TX), and converted to cDNA as described [28].

For gene expression analysis by OLG, brains from WT and GFAPγR1Δ at d30 p.i. were homogenized using a Papain Neuronal Tissue Dissociation Kit (Miltenyi, Auburn, CA). Cell pellets from homogenates were resuspended in RPMI medium containing 25 mM HEPES (pH 7.2), adjusted to 30% Percoll, and purified from a discontinuous 30/70% Percoll gradient as described above. Cells were washed in RPMI medium and blocked prior to staining as described above. Cells were then stained with anti-CD45 (clone 30-F11, BD Biosciences) APC, unconjugated O4 mAb, and anti-mouse IgM PE (clone R6-60.2) (BD Biosciences) for 30 min and OLG-purified by FACS based on their CD45^−^ O4^+^ phenotype as described [59, 60] using a FACS/ARIA cell sorter (BD Biosciences). OLG were resuspended in 450 μl of Trizol reagent (Invitrogen), and RNA was extracted using the Direct-zol RNA mini-prep kit (Zymo, Irvine, CA) following the manufacturer’s instructions. ET-1 (Mm00438656_m1), TNFR1 (Mm00441883_g1), and TNFR2 (Mm00441889_m1) messenger RNA (mRNA) levels were measured by quantitative real-time PCR using the Taqman Gene Expression Assay and Taqman primers (Life Technologies, Carlsbad, CA). Gene expression analysis was performed using a 7500 Fast real-time PCR System (Applied Biosystems, Foster City, CA).

### Statistical analysis

Data represent the mean ± SEM. Significance was determined using two-tailed Student’s *t* test or Wilcoxon rank sum-test using SigmaStat V3.5. A value of *P* < 0.05 was considered statistically significant. Graphs were plotted using GraphPad Prism v5.02 software. For IHC analysis, data is presented from two to three separate fields per mouse with a minimum of three mice per group and time point and experiment. Two independent experiments were evaluated for each analysis. For RT-PCR analysis, spinal cords from four to five mice per group were used.

## Results

### TNF neutralization reduces demyelinating lesion size during progressive EAE

**Fig. 1:**
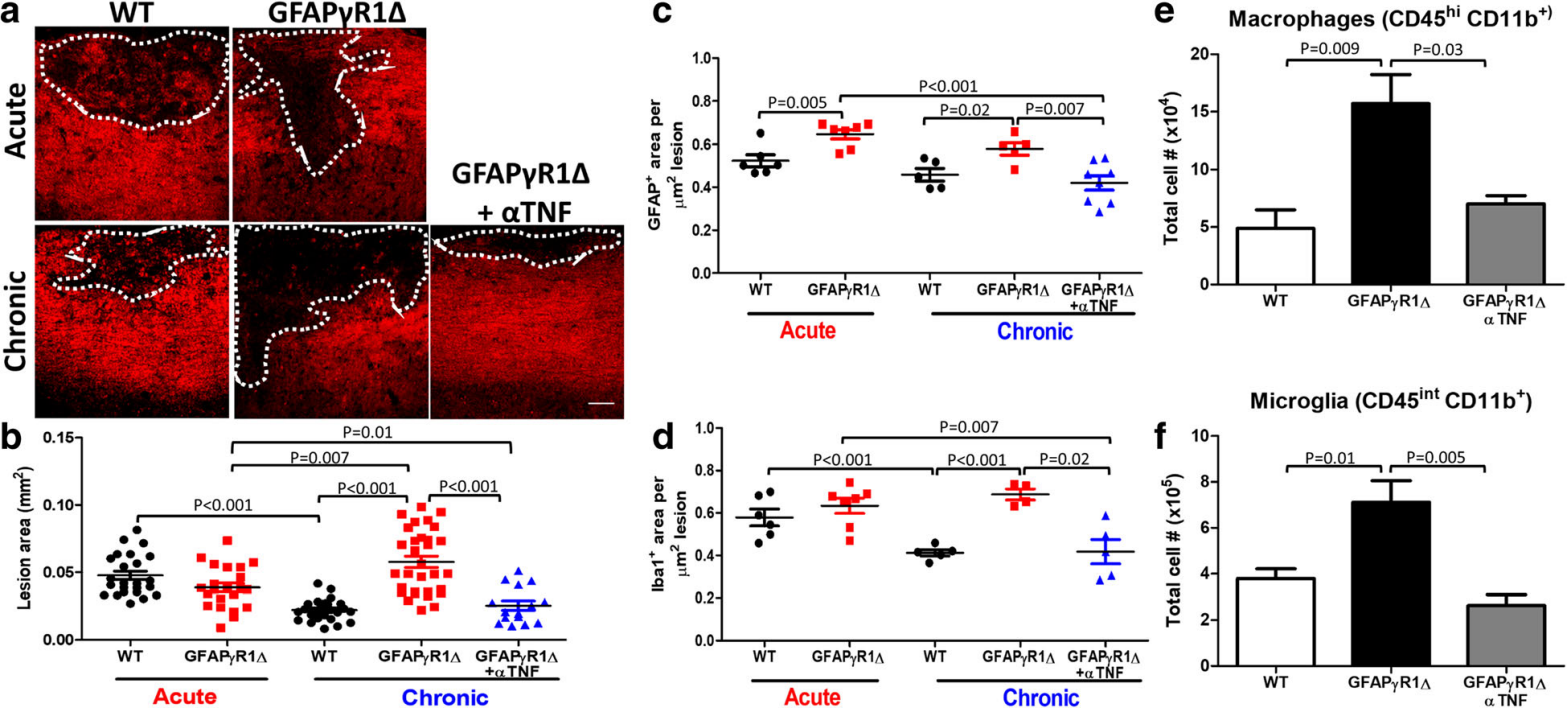
TNF blockade limits demyelinating lesion size coincident with ameliorated astrocyte and myeloid cell reactivity during progressive EAE in GFAPγR1Δ mice. **a** Representative confocal Z-stack images depicting fluoromyelin staining of longitudinal spinal cord sections from WT and GFAPγR1Δ mice treated either with isotype control (GFAPγR1Δ) or anti-TNF mAb (GFAPγR1Δ + αTNF) during acute (d19 p.i.) and chronic EAE (d30 p.i.). Demyelinated areas identified by loss of fluoromyelin staining are delineated by dotted white line. Scale bar, 50 μm. **b** Quantification of lesion size (mm^2^) per field of view in WT (black), GFAPγR1Δ (red), and GFAPγR1Δ + αTNF (blue) mice during acute (d19 p.i.) and chronic EAE (d30 p.i.). Each symbol represents one lesion, with *n* compiled from three to four fields per mouse with five to six mice per group from two to three independent experiments. **c** Astrocyte (GFAP) and **d** macrophage/microglia (Iba1) positive area per square micrometer demyelinating lesion area. *n* = 3–4 fields per mouse with 2–3 mice per group. Total numbers of **e** macrophages (CD45^hi^ CD11b^+^) and **f** microglia (CD45^int^ CD11b^+^) in the CNS of all three mouse groups at d30 p.i. assessed by flow cytometry. Data represents mean ± SEM from pooled samples of five mice per group per experiment, three to four independent experiments per group. *P* values were determined by Student’s *t* test. GFAPγR1Δ in all panels represents GFAPγR1Δ mice treated with isotype control mAb

Monoclonal antibody (mAb)-mediated TNF blockade in GFAPγR1Δ mice developing progressive EAE was previously shown to be therapeutic as evidenced by substantially reduced clinical disease, inflammation, and demyelination [28]. To assess whether TNF neutralization not only limits ongoing demyelination but also promotes tissue repair, we measured demyelinating lesion size during acute (d19 p.i.) and chronic (d30 p.i.) EAE in spinal cords of both WT and GFAPγR1Δ mice (Fig. 1a, b). In WT mice, lesion size decreased by ~ 2-fold during disease remission, implying tissue repair and remyelination. Although lesion size in GFAPγR1Δ mice undergoing acute EAE was slightly reduced compared to WT mice, it was significantly increased during disease progression, consistent with previous results [26–28]. Anti-TNF treatment of GFAPγR1Δ diminished lesion size by ~ 2.5-fold compared to isotype mAb-treated controls. Moreover, lesion size shrank by 50% compared to the acute phase suggesting that TNF blockade in GFAPγR1Δ not only limits lesion progression but also promotes repair.

Tissue damage is associated with astrogliosis and microglia/macrophage activation as indicated by thickening of astrocytic extensions and mesh formation [21, 24], as well as retraction and thickening of microglia/macrophage protrusions [61]. Spinal cords during acute and chronic EAE from all groups were therefore stained for fluoromyelin and GFAP or Iba1 reactivity to determine the extent of astrocyte and macrophage/microglia activation within lesions (Additional file 1). Astrogliosis within demyelinated areas was significantly increased in GFAPγR1Δ mice during both acute and chronic disease compared to WT mice (Fig. 1c). However, GFAP reactivity within each mouse group was similar between the acute and chronic phase. Contrasting GFAP reactivity, no differences were evident in myeloid cell reactivity during acute EAE comparing WT and GFAPγR1Δ mice (Fig. 1d). However, while Iba1 reactivity decreased within the lesions during disease remission in WT mice, it remained elevated within lesions during progressive EAE in GFAPγR1Δ mice (Fig. 1d). Nevertheless, both GFAP and Iba1 reactivity in GFAPγR1Δ mice were significantly ameliorated by TNF blockade relative to isotype mAb-treated controls as well as mice in the acute EAE phase. Increased macrophage/microglia reactivity correlated with elevated numbers of both monocyte-derived CD45^hi^ CD11b^+^ macrophages (Fig. 1e) and CD45^int^ CD11b^+^ microglia (Fig. 1f) in the CNS of GFAPγR1Δ, which were reduced to levels in remitting WT mice by anti-TNF treatment. These data suggest that mechanisms underlying repair in remitting WT mice are dysregulated in GFAPγR1Δ mice due to increased astrogliosis already evident in lesions during the acute phase and maintained during chronic disease. Increased astrogliosis and myeloid reactivity is mitigated by neutralization of excessive TNF.

### TNF blockade reduces OLG apoptosis during progressive EAE

**Fig. 2:**
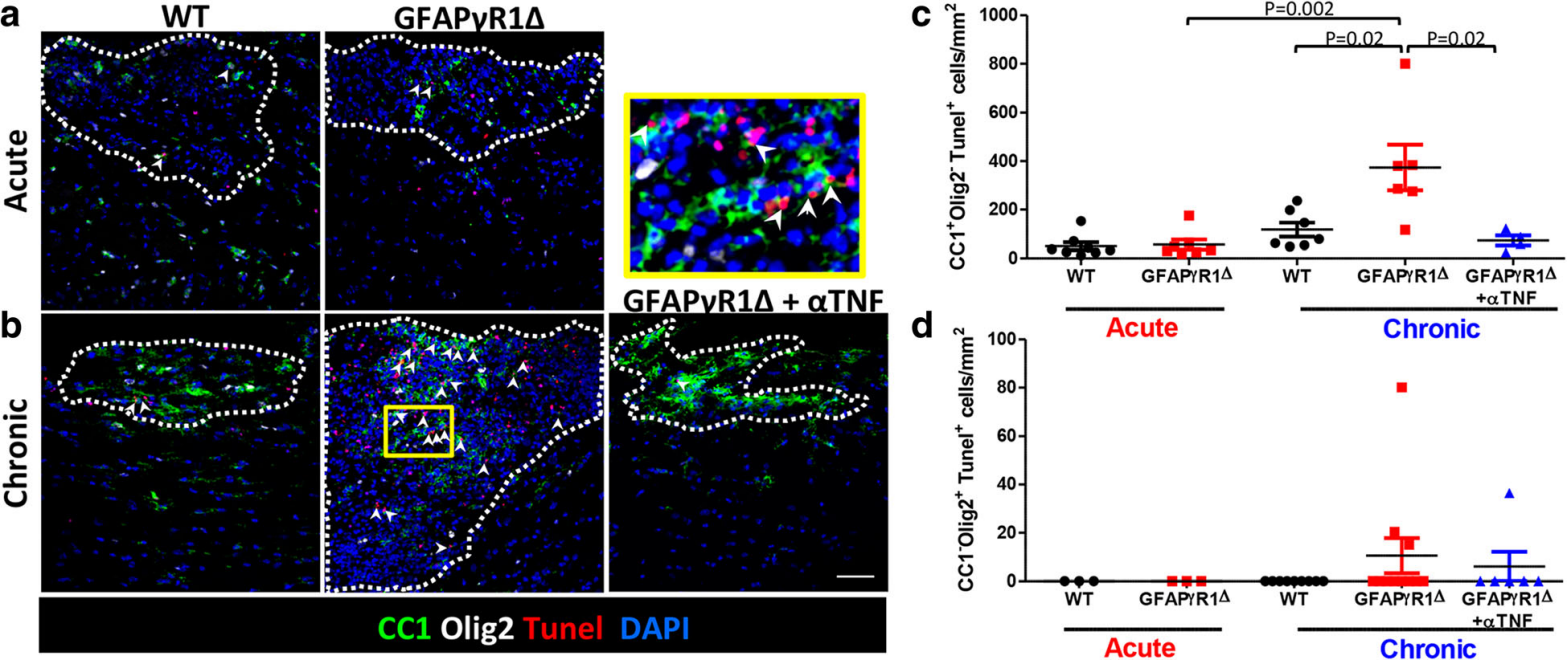
TNF neutralization limits OLG apoptosis during progressive EAE in GFAPγR1Δ mice. Confocal Z-stack images showing CC1 (mature OLG, green), TUNEL (red), Olig2 (white), and DAPI (blue) staining within spinal cord demyelinating lesions (dotted line) during **a** acute (d19 p.i.) and **b** chronic (d30 p.i.) EAE in WT and GFAPγR1Δ mice, treated with isotype control or anti-TNF mAb. White arrows indicate TUNEL^+^ mature CC1^+^Olig2^−^ cells. Scale bar, 50 μm. The area highlighted in yellow is shown at × 100-fold magnification to better visualize apoptotic cell types. **c** Quantification of apoptotic myelinating OLG (TUNEL^+^ CC1^+^Olig2^−^) per square millimeter lesion area. **d** Quantification of apoptotic OPC (TUNEL^+^ CC1^−^Olig2^+^) OPC per square millimeter lesion area. Data in **c** and **d** represent mean values ±SEM of two to three separate fields per mouse with two to three mice per group from two independent experiments. *P* values were determined by Student’s *t* test. GFAPγR1Δ in all panels represents GFAPγR1Δ mice treated with isotype control mAb

TNF has been implicated in both promoting demyelination by inducing mature OLG apoptosis [36–39, 41, 42] and inhibiting OPC differentiation [40]. To address whether sustained TNF in GFAPγR1Δ mice enhances OLG apoptosis during progressive EAE, spinal cord sections were stained for CC1 expression to mark mature myelinating OLG and OLIG2 to mark OLG lineage cells and for TUNEL reactivity to identify apoptotic cells. During acute EAE, apoptotic mature myelinating OLG (CC1^+^Olig2^−^ TUNEL^+^) per square millimeter lesion area were comparable between WT and GFAPγR1Δ mice (Fig. 2a, c). The number remained similarly low during disease remission in WT mice, but increased by ~ 4-fold/mm^2^ lesion area in GFAPγR1Δ mice with progressive EAE (Fig. 2b, c). Elevated apoptotic OLG at d30 p.i. in GFAPγR1Δ correlated with enlarged lesions (Fig. 1a, b) and increased cellular density determined by DAPI staining. TNF blockade significantly reduced apoptotic OLG per square millimeter lesion area to levels in WT mice. OPC (CC1^−^ Olig2^+^ TUNEL^+^) apoptosis per square millimeter lesion area remained sparse in all groups (Fig. 2d). Sustained TNF thus correlated with increased lesion size and increasing OLG death consistent with accumulating adverse effects of TNF. The numbers of apoptotic OLG in non-lesioned areas were significantly lower (~ 5-fold less) relative to lesioned areas during acute EAE with no evident differences between both groups (Additional file 2A). However, GFAPγR1Δ mice with progressive EAE exhibited a ~ 2-fold increase in OLG apoptosis relative to remitting WT mice. TNF neutralization also resulted in a ~ 2-fold reduction of apoptotic OLG in non-lesioned areas. These results demonstrate that TNF neutralization prevents increasing OLG death during progressive EAE and that OPC are spared from TNF toxicity in this transgenic model.

While the majority of TUNEL^+^ cells within lesions were OLG, a significant number did not co-stain with CC1 (Fig. 2a, b). As EAE is also characterized by prominent T cell apoptosis [62–64], and T cells remain elevated during progressive EAE in GFAPγR1Δ mice [26–28], we also assessed potential differences in T cell apoptosis within demyelinating lesions. Apoptotic T cells per square millimeter lesion area reached about 50% of apoptotic OLG numbers during acute EAE at d19 p.i., with no differences between the WT and GFAPγR1Δ mice (Additional file 2B). However, at d30 p.i., TUNEL^+^ T cells were increased ~ 8-fold in WT and ~ 10-fold GFAPγR1Δ relative to their respective levels in the acute phase. Comparing apoptotic T cells in the chronic phase, GFAPγR1Δ mice had ~ 2-fold increased numbers than WT mice, which were reduced to WT levels by anti-TNF treatment. Overall, these data support that OLG are the primary cells undergoing apoptosis within demyelinating lesions during progressive EAE in GFAPγR1Δ mice.

### Anti-TNF treatment promotes OPC differentiation during progressive EAE

**Fig. 3:**
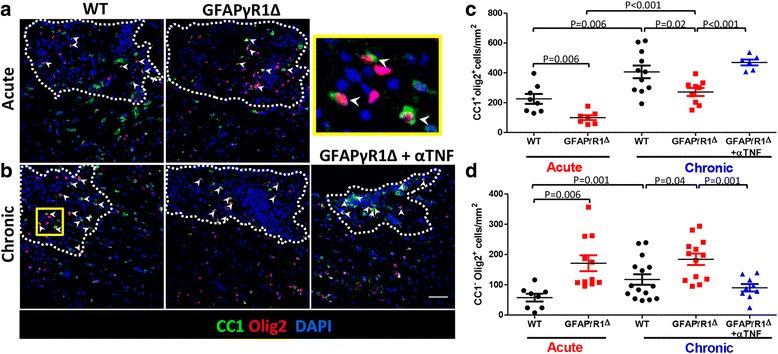
TNF blockade promotes OPC differentiation during progressive EAE in GFAPγR1Δ mice. Representative confocal Z-stack images showing CC1 (green), OLIG2 (red), and DAPI (blue) staining within spinal cord demyelinating lesions (dotted line) during **a** acute (d19) and **b** chronic (d30) EAE in WT and GFAPγR1Δ mice, treated with isotype control or anti-TNF mAb. White arrows indicate differentiated myelinating CC1^+^Olig2^+^ cells. Scale bar, 50 μm. **c** Quantification of differentiated OLG (CC1^+^Olig2^+^) per square millimeter lesion area. **d** Quantification of OPCs (CC1^−^Olig2^+^) per square millimeter lesion area. Data represent the mean values ±SEM from five to seven separate fields per mouse with two to three mice per group of two independent experiments. *P* values were determined by Wilcoxon rank sum-test. GFAPγR1Δ in all panels represents GFAPγR1Δ mice treated with isotype control mAb

In addition to mediating OLG apoptosis, TNF impairs OPC differentiation through a mitochondria-dependent metabolic process [40]. However, TNFR2 signaling promotes OPC differentiation, questioning the relative balance of both effects during a given disease stage. Since TNF blockade appears to promote repair in GFAPγR1Δ mice as indicated in Fig. 1, we assessed the effects of TNF neutralization on OPC differentiation using the dual CC1^+^Olig2^+^ phenotype to distinguish differentiated myelinating OLG from more mature CC1^+^Olig2^−^ OLG [55, 57, 58]. Spinal cords were stained for CC1 and Olig2 expression in combination with DAPI at 19 and 30 days p.i. and CC1^+^Olig2^+^ OLG quantified per square millimeter lesion area. Despite similar lesion size between WT and GFAPγR1Δ during acute EAE, differentiated OLG were surprisingly lower in GFAPγR1Δ mice (Fig. 3a, c). During disease remission in WT mice, CC1^+^Olig2^+^ OLG per square millimeter lesion area increased ~ 2-fold (Fig. 3a–c). Although CC1^+^Olig2^+^ OLG also increased ~ 2-fold between the acute and progressive time points in GFAPγR1Δ mice, they remained reduced by ~ 1.5-fold compared to WT mice undergoing remission. Importantly, anti-TNF mAb treatment raised the numbers of CC1^+^Olig2^+^ OLG to those observed in WT lesions. These data suggest that TNF neutralization overcomes a block in OPC maturation already evident in the acute EAE disease phase in GFAPγR1Δ mice. CC1^+^Olig2^+^ OLG numbers within non-lesioned areas were ~ 2-fold lower than within lesions in both acute and chronic EAE in WT mice (Additional file 3A). However, in GFAPγR1Δ mice, CC1^+^Olig2^+^ OLG numbers were similar between lesioned and non-lesioned areas at d19 and d30 p.i. TNF neutralization did not affect differentiated CC1^+^Olig2^+^ OLG numbers within non-lesioned areas.

To assure that impaired OPC differentiation in lesions during progressive EAE is not due to reduced OPC recruitment, the number of OPC (CC1^−^Oig2^+^) per square millimeter lesion area was determined. GFAPγR1Δ mice harbored ~ 3-fold higher numbers of OPCs than WT mice during acute EAE (Fig. 3d). While OPCs per lesion area increased by ~ 2-fold during remission in WT mice, they remained similar to the acute phase in GFAPγR1Δ mice during disease progression. Overall, OPC numbers in GFAPγR1Δ mice were ~ 2-fold higher than in WT mice during chronic EAE, but significantly reduced by TNF blockade, supporting their differentiation to myelinating OLG (Fig. 3d). OPC numbers in non-lesioned white matter were similar during acute EAE in both groups, but ~ 3-fold higher per square millimeter lesion than non-lesioned areas in GFAPγR1Δ mice (Additional file 3B). Conversely, in chronic EAE, OPCs were ~ 1.6-fold increased within demyelinated lesions relative to non-demyelinated areas in WT mice, while no differences were noted in GFAPγR1Δ mice with progressive EAE; comparison of OPCs in non-lesioned areas of GFAPγR1Δ mice relative to WT mice showed a ~ 2-fold increase. Anti-TNF treatment also reduced the OPCs in non-lesioned areas correlating with the increase in OPC differentiation. These data suggest that progressive EAE in GFAPγR1Δ mice is not only associated with increased OLG apoptosis, but also a defect in OPC differentiation, which is overcome by TNF blockade.

### TNFR2 mRNA expression is reduced during progressive EAE

**Fig. 4:**
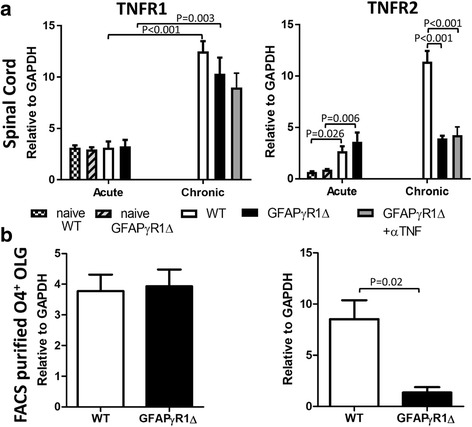
Reduced TNFR2 mRNA expression in OLG during progressive EAE in GFAPγR1Δ mice. **a** TNFR1 and TNFR2 mRNA levels determined by RT-PCR of total spinal cord RNA from WT and GFAPγR1Δ mice undergoing EAE at d19 and d30 p.i. Data represent the mean ± SEM of individual mice from two separate experiments, each composed of three to four mice per group. **b** TNFR1 and TNFR2 mRNA expression in FACS-purified O4^+^ OLG at d30 p.i assessed by RT-PCR. Data represent mean ± SEM from three individual experiments, each comprising six pooled brains. *P* values determined by one-way ANOVA. GFAPγR1Δ in all panels represents GFAPγR1Δ mice treated with isotype control mAb

Enhanced OPC differentiation and remyelination by total TNF blockade in our progressive EAE model is inconsistent with numerous reports demonstrating beneficial TNFR2-mediated effects in promoting OPC differentiation [17, 44–49, 65]. This apparent discrepancy suggested that elevated and sustained TNF during progressive EAE acts primarily via detrimental TNFR1 signaling without an evident protective TNFR2 component. To assess whether protective TNF/TNFR2-mediated effects are overwhelmed and/or if TNFR2 is dysregulated, we determined TNFR1 and TNFR2 mRNA expression during progressive EAE in total spinal cord RNA (Fig. 4a). EAE in WT and GFAPγR1Δ mice did not alter TNFR1 mRNA levels compared to naïve mice at d19 p.i. Moreover, although TNFR1 mRNA levels were upregulated at day 30 p.i., they remained comparable among groups. TNFR2 mRNA levels were elevated in both groups relative to naïve mice during acute EAE. However, while WT mice undergoing disease remission strongly upregulated TNFR2 mRNA, GFAPγR1Δ mice with progressive EAE exhibited no increase in TNFR2 mRNA expression. Moreover, TNF blockade did not overcome the apparent block in TNFR2 mRNA upregulation.

Since the overall TNFR2 expression is reduced during progressive EAE and TNFR2 signaling on OLG is important for OLG survival and function, TNFR2 mRNA expression was also specifically evaluated in FACS sorted O4^+^ OLG at d30 p.i. (Fig. 4b). TNFR2 mRNA levels were indeed ~ 6-fold lower in O4^+^ OLG derived from GFAPγR1Δ than from WT mice. Reduced TNFR2 mRNA expression in OLG correlates with increased OLG apoptosis and reduced OPC differentiation during progressive EAE. No difference in TNFR1 mRNA between WT and GFAPγR1Δ was observed. These data support the notion that excessive TNF preferentially acts through TNFR1 due to an imbalance between TNFR1/TNFR2 signaling. Importantly, these results are consistent with no evident negative effects of antibody-mediated neutralization of both TNF forms in GFAPγR1Δ mice.

### TNF neutralization reduces endothelin-1 (ET-1) levels during progressive EAE

The overriding detrimental TNF effects during progressive EAE suggested additional mediators may impair OPC differentiation and mask TNFR2-mediated repair in GFAPγR1Δ mice. A review of the literature revealed ET-1, a potent vasoactive peptide produced by endothelial cells and reactive astrocytes [54, 66], as a potential candidate affecting demyelinating disease. ET-1 promotes reactive astrogliosis in both MS and EAE and exacerbates clinical disease in EAE [54, 67–70]. Overexpression of ET-1 by astrocytes results in severe EAE [67], while blocking ET-1 signaling ameliorates disease [68]. A focal demyelination model further revealed that ET-1 released by reactive astrocytes inhibits OPC differentiation [55]. Critical to the present studies, TNF stimulation of ET-1 is reciprocal in various non-CNS diseases [71, 72].

**Fig. 5:**
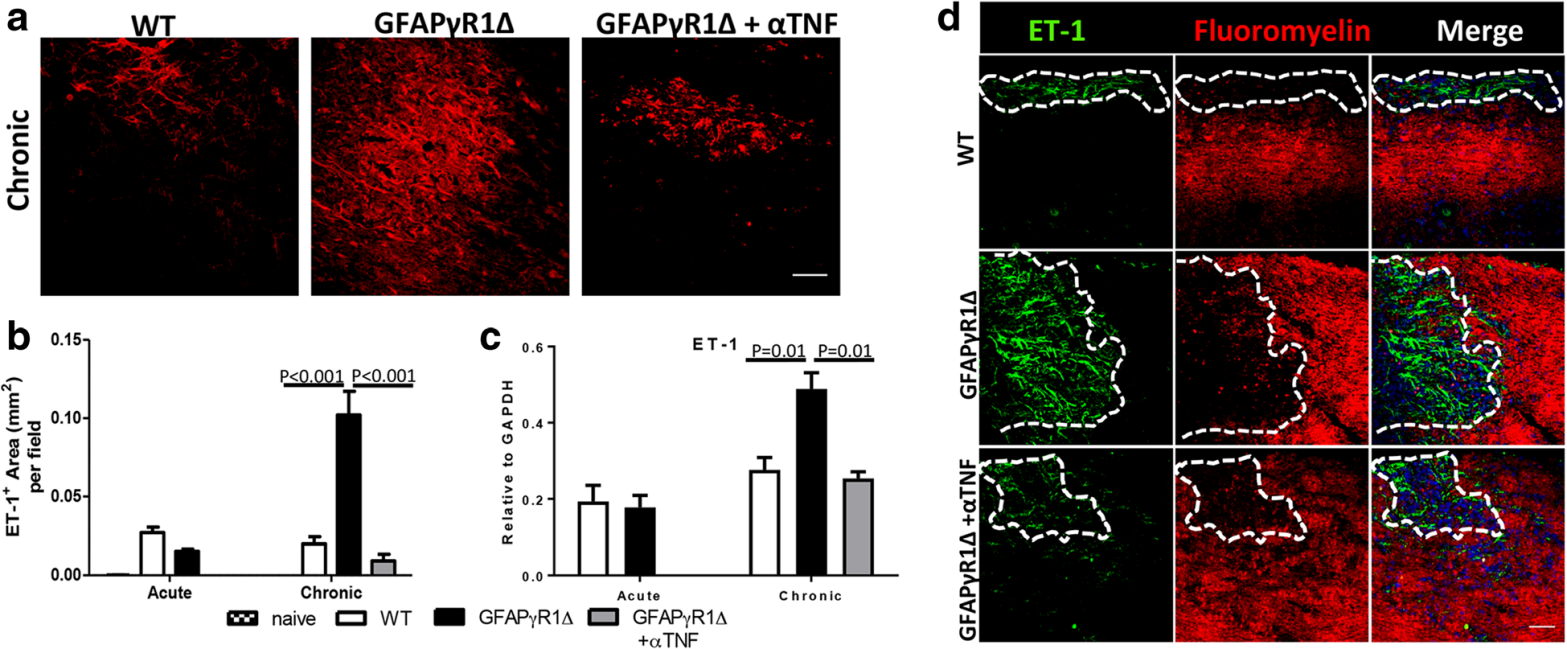
TNF blockade limits ET-1 expression during progressive EAE. **a** Representative confocal images (Z-stack) of longitudinal spinal cord sections stained for ET-1 during chronic (d30) EAE in WT and GFAPγR1Δ mice, treated with isotype control or anti-TNF mAb. **b** Quantification of ET-1^+^ area per field (mm^2^) in spinal cords stained as indicated in panel **a** during acute (d19) and chronic (d30) EAE. Data are from six to eight separate fields from two individual mice per group and two independent experiments. **c** ET-1 mRNA levels in spinal cords from EAE mice as indicated determined by RT-PCR. Data represent mean ± SEM values from individual mice of two separate experiments, each comprising three to four mice per group and time point. **d** Representative confocal images (Z-stack) of spinal cords from WT and GFAPγR1Δ mice, treated with isotype control or anti-TNF mAb, during chronic (d30) EAE stained for ET-1 (green), fluoromyelin (red), and DAPI (blue) within demyelinated lesion (dotted line). Images representative of three to four separate fields from two to three mice per group. Scale bars, 50 μm. *P* values were determined by Student’s *t* test. GFAPγR1Δ in all panels represents GFAPγR1Δ mice treated with isotype control mAb

To better understand mechanisms underlying limited OPC differentiation during progressive EAE, we compared ET-1 expression in spinal cords during acute and chronic EAE phases in WT and GFAPγR1Δ mice (Fig. 5a, b). ET-1 immunoreactivity was minimal in spinal cords from naïve WT and GFAPγR1Δ mice (data not shown). During EAE, ET-1 localization was restricted to defined areas consistent with lesions in both groups. While no differences in expression were noted during acute EAE, GFAPγR1Δ mice exhibited a ~ 5-fold increase in ET-1 immunoreactivity per field of view compared to the acute phase as well as to WT mice in the chronic phase (Fig. 5a, b). By contrast, ET-1 levels in remitting WT mice remained constant or slightly decreased relative to the acute phase. Importantly, TNF blockade significantly reduced ET-1 to levels similar to or below those in WT mice. RT-PCR analysis of ET-1 mRNA levels in spinal cords during acute and chronic EAE supported the histology results (Fig. 5c). ET-1 is highly produced by reactive astrocytes within demyelinated areas in MS and experimental lysolecithin-induced demyelination [54, 55, 66]. To confirm that ET-1 expression is mainly restricted to lesions, we performed co-staining with anti-ET-1 mAb and fluoromyelin. Over 90% of ET-1 was localized to demyelinated lesions in all EAE groups (Fig. 5d).

### ET-1 primarily colocalizes with lesion-associated myeloid cells and reactive astrocytes during progressive EAE

**Fig. 6:**
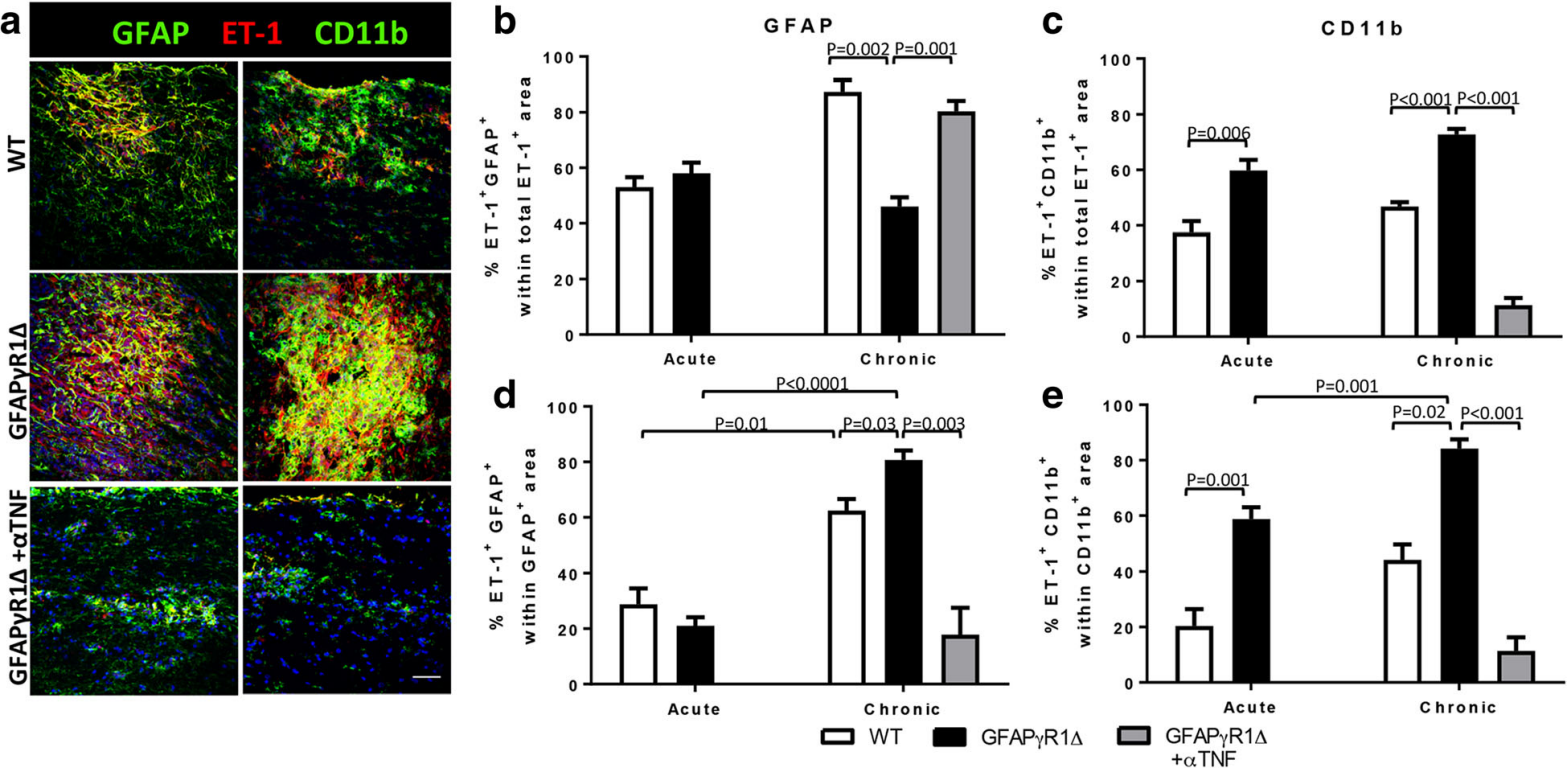
ET-1 colocalizes with reactive astrocytes and myeloid cells during progressive EAE in GFAPγR1Δ mice. **a** Representative confocal images (Z stack) of spinal cords from WT and GFAPγR1Δ mice, treated with isotype control or anti-TNF mAb, during chronic (d30) EAE stained for ET-1 (red), DAPI (blue), and either GFAP to identify astrocytes (left, green), or CD11b to identify macrophages/microglia (right, green), Scale bars, 50 μm. Images are representative of three to four separate fields from two to three mice per group. The distribution of ET-1 reactivity among astrocytes (**b**) or myeloid cells (**c**) was quantified by dividing the dually ET-1^+^ GFAP^+^ staining area by the total ET-1^+^ area per field or the ET-1^+^ CD11b^+^ staining area by the total ET-1^+^ area per field, respectively, in the fields described under panel **a**. Values are plotted as percentages of ET-1^+^ GFAP^+^ or ET-1^+^ CD11b^+^ reactivity within total ET-1 reactivity in panels **b** and **c**, respectively. Conversely, the percent of astrocytes (**d**) or myeloid cells (**e**) expressing ET-1 was assessed by dividing the ET-1^+^ GFAP^+^ or ET-1^+^ CD11b^+^ dual staining area by the **e** total GFAP^+^ or CD11b^+^ area within demyelinated lesions. Data represent mean ± SEM. *P* values were determined by one-way ANOVA on rank-sum test. GFAPγR1Δ in all panels represents GFAPγR1Δ mice treated with isotype control mAb

ET-1 is constitutively and inducibly expressed in a variety of cell types, including endothelial cells, reactive astrocytes, and myeloid cells [66, 71, 73]. The dominant source of increased ET-1 during progressive EAE was thus assessed by colocalization of ET-1 reactivity with GFAP (astrocytes) or CD11b (macrophages and microglia) (Fig. 6a). The relative proportion of ET-1 colocalizing with astrocytes or myeloid cells was quantified by dividing the area dually positive for ET-1 and GFAP (Fig. 6b) or ET-1 and CD11b (Fig. 6c) staining, respectively, by the total ET-1^+^ area per field of view. During acute EAE in both WT and GFAPγR1Δ mice, ET-1 expression distributed fairly evenly between astrocytes and myeloid cells with a slightly higher proportion colocalizing to astrocytes in WT mice (Fig. 6b, c). However, the ratios diverged significantly comparing remitting versus chronic EAE. While almost 80% of ET-1 colocalized with astrocytes and ~ 40% with myeloid cells in remitting WT EAE, the ratio was reversed in GFAPγR1Δ mice with less than 50% of ET-1 localizing to astrocytes and almost 80% to myeloid cells. The observation that relative percentages are above 100% of total was attributed to the density and spatial proximity of GFAP and CD11b staining within lesions. TNF blockade in GFAPγR1Δ mice not only reduced total levels of ET-1, but also reverted the proportion of ET-1 expression in astrocytes to ~ 80% and reduced the fraction of ET-1 colocalizing with CD11b to ~ 10% of total ET-1 area. Together, these data suggest that ET-1 is prominently associated with reactive astrocytes during EAE disease remission in WT and anti-TNF-treated GFAPγR1Δ mice but is significantly increased in myeloid cells during aggravated progressive disease in control GFAPγR1Δ mice.

In addition to evaluating the relative distribution of ET-1 expression among astrocytes and myeloid cells, we also assessed whether the proportion of astrocytes or myeloid cells expressing ET-1 within lesions was altered. This was achieved by dividing either the dually reactive GFAP^+^ ET-1^+^ or CD11b^+^ ET-1^+^ area by the total GFAP^+^ or CD11b^+^ area, respectively, per region of interest (Fig. 6d, e). Analysis of the acute disease phase revealed ~ 20–25% of astrocytes colocalized with ET-1 with no difference between WT and GFAPγR1Δ mice. In the chronic phase, the fraction of ET-1^+^ astrocytes increased to ~ 60% in WT and 80% in GFAPγR1Δ mice, revealing that ET-1 expression by astrocytes within demyelinated areas was significantly elevated in both groups (Fig. 6d). Interestingly, anti-TNF treatment reduced the proportion of ET-1^+^ astrocytes within lesioned areas to less than 20% resembling levels during the acute phase. The proportion of CD11b^+^ area co-staining with ET-1 was already increased by 3-fold during acute EAE in GFAPγR1Δ relative to WT mice. Moreover, during the chronic phase, the fraction of the dually staining ET-1^+^CD11b^+^ area rose to ~ 40% in WT and ~ 80% in GFAPγR1Δ mice (Fig. 6c). TNF blockade dramatically reduced the proportion of ET-1-expressing myeloid cells to less than 20%. These results indicated TNF blockade is associated with a significant reduction in ET-1 expression by both astrocytes and myeloid cells, which correlates with reduced astrocyte and myeloid cell reactivity within lesions observed in Fig. 1c, d.

### Endothelin-1 receptor B expression is increased during progressive EAE

**Fig. 7:**
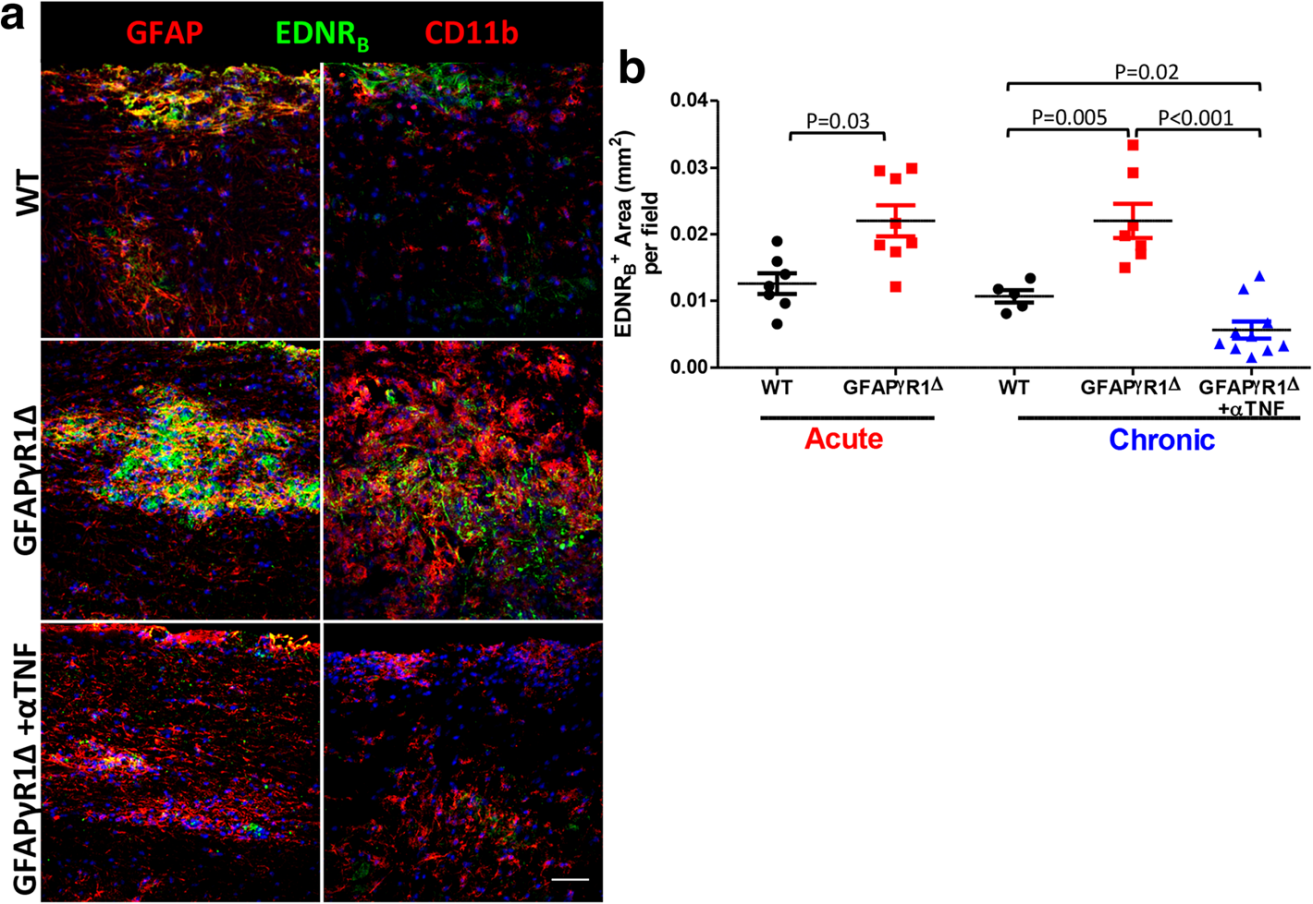
TNF blockade reduces EDNR_B_ expression during progressive EAE in GFAPγR1Δ mice. **a** Confocal images (Z-stack) of spinal cords stained for EDNR_B_ (green), DAPI (blue), and GFAP (astrocytes, red) or CD11b (macrophages/microglia, red) in WT, GFAPγR1Δ and GFAPγR1Δ + αTNF at d30 p.i. Images are representative of six to eight separate fields from two individual mice per group and two individual experiments. Scale bar, 50 μm. **b** Graph showing EDNR_B_ positive area (mm^2^) per field at d19 and d30 p.i. Data represent mean ± SEM. *P* values determined by Wilcoxon rank-sum test. GFAPγR1Δ in all panels represents GFAPγR1Δ mice treated with isotype control mAb

The deleterious effects of ET-1 are primarily mediated by ET-1 signaling through ET-1 receptor B (EDNR_B_) on reactive astrocytes [74]. To assess EDNR_B_ levels and identify cells expressing EDNR_B_ during progressive EAE, spinal cords were stained for EDNR_B_ and GFAP or CD11b expression. EDNR_B_ primarily colocalized with GFAP in both WT and GFAPγR1Δ at d19 and d30 p.i (Fig. 7a, b). Colocalization of EDNR_B_ with CD11b was minimal in WT mice but increased during progressive EAE. In anti-TNF-treated GFAPγR1Δ mice, EDNR_B_ primarily colocalized with GFAP and only sparsely with CD11b. EDNR_B_ immunostaining in naïve mice was near detection levels (data not shown). During acute and chronic EAE, the EDNR_B_^+^ area per field of view was ~ 2-fold higher in GFAPγR1Δ than in WT mice and reduced by ~ 4-fold following anti-TNF treatment (Fig. 7b). EDNR_B_ mRNA levels were ~ 3-fold higher in progressive EAE in GFAPγR1Δ mice than subsiding EAE in WT mice; however, TNF neutralization did not alter EDNR_B_ mRNA levels (data not shown). EDNR_A_ mRNA levels were similar among all groups and did not change from naïve mice (data not shown).

## Discussion

TNF blockade is therapeutic in several progressive autoimmune diseases such as rheumatoid arthritis, ankylosing spondylitis, and inflammatory bowel disease. However, despite strong preclinical data supporting TNF neutralization as a therapeutic for MS, relapsing remitting patients treated with lenercept, a soluble TNFR1 IgG fusion protein, developed increased relapse rates and worsening of neurological functions [43]. Similarly, treatment of two rapidly progressing MS patients with anti-TNF mAb exacerbated disease consistent with augmented lesion numbers and lymphocyte infiltration [75]. Conversely, treatment of primary and secondary P-MS patients with the TNF inhibitor pirfenidone improved clinical disability [76, 77]. These opposing results in MS may be attributed to the dual functions of TNF engagement of TNFR1 and TNFR2 in CNS inflammation and demyelination, as well as disease severity. EAE studies in WT mice demonstrated that TNF blockade in early stages of disease ameliorates or delays disease onset [78], while it worsens disease when initiated at the peak of acute EAE [46]. The implication that TNF contributes to tissue damage through TNFR1 at early disease stages but conversely exerts protective effects through TNFR2 during chronic EAE was confirmed in respective TNFR knockout mice [48, 52, 53]. However, distinct from these studies, neutralization of both TNF forms in GFAPγR1Δ mice ameliorated disease; moreover, reduced demyelination coincided with reduced BBB permeability, reduced CNS cellular infiltration indiscriminate of cell type, and enhanced anti-inflammatory responses [28]. These findings suggested that detrimental TNF signals can override protective TNF/TNFR2 effects in some clinical settings during progressive disease. This was supported by continued lesion expansion and destruction in GFAPγR1Δ mice during chronic EAE, contrasting decreasing lesion size in WT mice. Importantly, anti-TNF treatment not only constrained lesion expansion, but also decreased lesion size compared to the acute phase, suggesting repair.

In vivo and in vitro, TNF/TNFR1 signaling leads to neuron and OLG death and increased chronic inflammation, whereas TNF/TNFR2 interactions increase cell survival, anti-inflammatory signals, and remyelination [44, 46–49]. Analysis of the mechanisms underlying increased lesion size during EAE in GFAPγR1Δ mice indicated sustained TNF both enhanced mature OLG death and inhibited OPC differentiation into myelinating OLG. Despite an increase in differentiated OLG during progressive EAE relative to the acute phase in GFAPγR1Δ mice, their numbers remained significantly lower than in WT mice. Notably, these differences could not be attributed to impaired OPC recruitment. Thus, although present, newly differentiated OLG in demyelinated areas during progressive EAE were insufficient to repair the extensive tissue damage. Importantly, anti-TNF treatment limited OLG death and also promoted remyelination independent of TNFR2. Despite the established influence of TNFR1 and TNFR2 signaling, the relative temporal regulation and dynamics of their function in various stages of demyelinating disease are less well defined. Our analysis of TNFR mRNA expression surprisingly revealed that TNFR2 but not TNFR1 mRNA levels were significantly reduced in the CNS of GFAPγR1Δ compared to those of WT mice during chronic EAE, although no differences in TNFR1 or TNFR2 mRNA were evident in naïve GFAPγR1Δ mice. Moreover, reduced TNFR2 mRNA expression in FACS-purified OLG from GFAPγR1Δ compared to WT mice suggested an imbalance in TNFR1 relative to TNFR2 signaling during progressive EAE counteracts repair, which may explain why neutralization of both TNF forms confers protection in GFAPγR1Δ but not WT mice. Increased mature OLG death observed in the GFAPγR1Δ mice during chronic EAE may be partially responsible for differential TNFR1 and TNFR2 expression. However, numerous other cells express TNFR2, including microglia, macrophages, and regulatory T cells (Treg). As TNFR2 signaling on these cells has been shown to contribute to repair via enhancing clearance of debris and Treg suppressor function [47, 58], a potential role of these cells awaits future analysis. Irrespectively, reduced TNFR2 expression in GFAPγR1Δ mice implies sustained elevated TNF over time preferentially signals through TNFR1 to mediate OLG apoptosis. These findings are reminiscent of the therapeutic effects of the TNF inhibitor pirfenidone in P-MS patients [76, 77] and suggest that dysregulated TNFR2 expression may contribute to P-MS.

Impaired OPC differentiation may also reside in TNFR2-independent functions mediated by ET-1. ET-1 has been demonstrated to indirectly inhibit OPC maturation by upregulating expression of jagged-1 in reactive astrocytes, which binds to notch-1 in OPCs [55]. While TNF induces ET-1 in a variety of cell types [71, 72, 79], specific induction in astrocytes has not been reported to our knowledge. The ability of ET-1 to promote TNF production [80] and TNF to induce ET-1 production [81] implicates an autonomous self-enhanced loop exacerbating OLG death and blocking repair. This scenario is indeed supported by a vast increase in ET-1 expression coincident with increased and sustained macrophage-derived TNF during disease progression in GFAPγR1Δ mice. The dramatic reduction in ET-1 by TNF blockade supports TNF in promoting ET-1 production. Similar to MS, ET-1 primarily localized to white matter lesions in both subsiding and progressive EAE. However, while ET-1 primarily colocalized with astrocytes during subsiding EAE in WT mice, it colocalized with both astrocytes and myeloid cells during progressive EAE in GFAPγR1Δ mice, implicating microglia/macrophages as an additional potent source of ET-1 during exacerbated demyelination. Whether ET-1 is primarily produced by monocyte-derived macrophages or microglia remains to be determined. Nevertheless, the increased population of monocyte-derived macrophages in the CNS of GFAPγR1Δ mice [28] implicates this population as the more prominent source analogous to TNF.

The relevant CNS cell types responding to ET-1 have been under investigation [54, 69, 82]. After CNS injury, the ET-1 receptor ENDR_B_ is upregulated in glial cells and specifically in reactive astrocytes during demyelination [83]. During lysolecithin-induced demyelination, ET-1 signals through EDNR_B_ in reactive astrocytes, thereby mediating inhibitory jagged-1/notch-1 signaling in OPCs [55, 74]. Moreover, loss of EDNR_B_ in astrocytes, but not OPC, promotes remyelination [74]. Our data confirm EDNR_B_ expression predominantly in reactive astrocytes in subsiding WT EAE. GFAPγR1Δ mice undergoing progressive EAE exhibited vastly increased EDNR_B_ expression, which was also mostly associated with astrocytes, although modest macrophage/microglia colocalization was also evident. Anti-TNF treatment of GFAPγR1Δ mice resulted in reduced astrocyte and macrophage/microglial activation coincident with reduced EDNR_B_ expression. As little is known about the role of ET-1 and EDNR_B_ expression by myeloid cells in CNS pathology, further analysis is essential to delineate the contribution of ET-1/EDNR_B_ in myeloid cells versus astrocytes in this model. Similar EDNR_A_ mRNA expression in naïve and EAE mice (data not shown) suggested EDNR_A_ does not play a role in progressive EAE. Overall, our study supports an inhibitory role of ET-1, EDNR_B_, and astrocytes in limiting repair.

ET-1 has several other functions contributing to CNS damage, including breakdown of BBB integrity, thereby promoting CNS entry of peripheral inflammatory cells [82]. It also induces astrocytic production of CCL2, a chemoattractant for inflammatory monocytes, as well as IL-1β, IL-6, and reactive oxygen species, factors all contributing to CNS cellular damage and dysfunction [80]. Lastly, it inhibits CX3CL1, which confers neuroprotection in several demyelinating models [84]. Progressive EAE in GFAPγR1Δ mice is indeed associated with increased BBB permeability and elevated indiscriminate leukocyte infiltration compared to remitting WT mice, which are both alleviated by TNF blockade [28]. On the other hand, ET-1 has been shown to induce transforming growth factor β (TGFβ) [85, 86] and an M2 phenotype in human macrophages in vitro [73], known mediators of protection during EAE. Nevertheless, no evidence for increased TGFβ [26] or M2 macrophage/microglia during progressive EAE dismiss ET-1 mediated anti-inflammatory effects in our model.

Several mechanisms independent of OLG apoptosis may further contribute to lesion expansion during EAE in GFAPγR1Δ mice. Apoptotic T cells may sustain cytokine release and contribute to tissue debris. During injury or insult, debris clearance triggers tissue regeneration and is an essential component for CNS homeostasis [87]. Insufficient debris clearance by microglia is highly prevalent in many neurodegenerative diseases and is associated with impaired remyelination. Increased apoptotic T cells observed in GFAPγR1Δ mice during progressive EAE may thus hinder tissue repair. Given the more reactive phenotype of macrophages relative to microglia during progressive EAE [27, 28], ET-1 and EDNR_B_ expression mostly restricted to macrophages may additionally sustain pro-inflammatory effects. Overall enhanced lesion activity was supported by significantly increased astrocyte and myeloid cell activation during progressive EAE, which was limited by anti-TNF treatment. Furthermore, in a model of viral-induced demyelination, we have recently uncovered that the anti-inflammatory cytokine IL-10 is critical in limiting expansion of demyelinating lesions by promoting glial mesh formation at lesion borders [88]. Interestingly, IL-10 is downregulated during progressive EAE, and TNF neutralization increases IL-10 production [28]. Although IL-10 is known to downregulate TNF production [89], it is not clear whether TNF suppresses IL-10. Elevated IL-10 after TNF blockade may thus contribute to limiting demyelination during progressive EAE.

## Conclusions

**Fig. 8:**
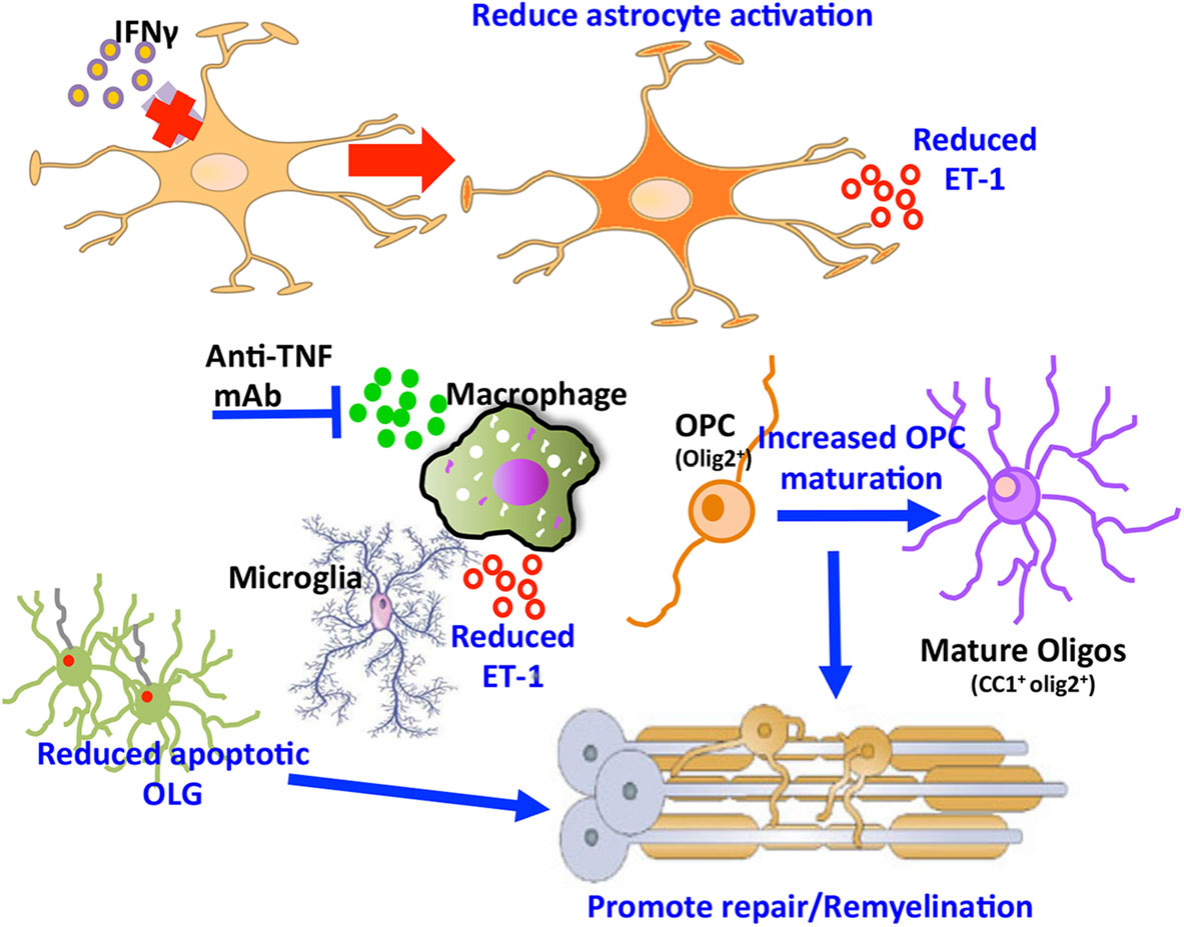
Model of astrocyte-dependent regulation of remyelination during progressive EAE. IFNγ signaling to astrocytes temporally coordinates the progression of inflammation and primes tissue for repair by limiting OLG apoptosis and promoting OPC differentiation. The absence of IFNγ-mediated anti-inflammatory responses in astrocytes increases astrogliosis and sustains TNF production by infiltrating monocytes, leading to elevated ET-1 expression in both astrocytes and myeloid cells. TNF/TNFR1-mediated interactions promote OLG death while ET-1/EdnRB signaling arrests OPC differentiation. TNF blockade reduces ET-1 and limits progressive demyelinating pathology

Overall, our results demonstrate that the inability of astrocytes to respond to IFNγ leads to progressive disability associated with sustained TNF production by macrophages, elevated ET-1 levels, and continued demyelinating lesion expansion. We propose a model in which a positive feedback loop sustaining TNF and ET-1 production exacerbates OLG apoptosis, inhibits OPC differentiation, and aggravates astrogliosis and myeloid cell activation in lesions. TNF blockade during sustained acute disease prevents ongoing lesion progression and severe clinical disability and promotes repair independent of TNFR2 (Fig. 8). The data further highlight that a better characterization of the interplay between TNF, ET-1, and regulation of their respective detrimental and beneficial receptors in the CNS promises to lead to more targeted therapies promoting remyelination and treating progressive forms of MS. More selective drugs targeting distinct TNF or ET-1 signaling pathways to alleviate demyelination have indeed been promising. For example, neutralization of sTNF using the sTNF specific inhibitor, Xpro1595, ameliorates EAE and promotes remyelination [46]. Similarly, sTNF blockade stimulates remyelination by enhancing myelin debris clearance by microglia during cuprizone-induced demyelination [47]. The EDNR_B_ inhibitor BQ-123 ameliorated EAE [68], and administration of a heterologous EDNR_B_ inhibitor, BQ-788, accelerated OPC differentiation during focal demyelination [55]. Dissecting mechanisms underlying benefits of selective TNF or ET-1 blockade in distinct demyelinating models thus shows promise to improve strategies promoting OPC differentiation following established demyelination and combat P-MS.

## Additional files

Additional file 1: TNF blockade reduces astrocyte and myeloid cell reactivity during progressive EAE in GFAPγR1Δ mice. Longitudinal spinal cord sections from WT and GFAPγR1Δ mice treated with isotype control or anti-TNF mAb were stained for astrocyte (GFAP) or myeloid cell (Iba-1) reactivity at d19 and d30 as indicated. Data represents three to four separate fields per mouse with two to three mice per group. (TIF 1843 kb)

Additional file 2: TNF neutralization limits OLG and T cell apoptosis during progressive EAE in GFAPγR1Δ mice. Longitudinal spinal cord sections from WT and GFAPγR1Δ mice, treated with isotype control or anti-TNF mAb, were stained for apoptosis during acute (d19) and chronic (d30) EAE. A. Quantification of apoptotic mature OLG (TUNEL^+^ CC1^+^ Olig2^−^) per square millimeter non-lesioned area. C. Quantification of apoptotic T cells (TUNEL^+^ CD3^+^) per square millimeter lesion area. Data represent mean ± SEM of two to three separate fields per mouse with two to three mice per group from two independent experiments. *P* values were determined by Student’s *t* test. GFAPγR1Δ in all panels represents GFAPγR1Δ mice treated with isotype control mAb. (TIF 133 kb)

Additional file 3: OPC maturation and recruitment is not impaired in non-lesioned areas during progressive EAE in GFAPγR1Δ mice. Longitudinal spinal cord sections from WT and GFAPγR1Δ mice, treated with isotype control or anti-TNF mAb, were stained for OLG and OPC during acute (d19) and chronic (d30) EAE. A. Quantification of differentiated myelinating OLG (CC1^+^Olig2^+^) per square millimeter non-lesioned area. D. Quantification of OPCs (CC1^−^Olig2^+^) per square millimeter non-lesioned area. Data represent the mean ± SEM of five to seven separate fields per mouse with two to three mice per group from two independent experiments. *P* values were determined by Wilcoxon rank sum-test. GFAPγR1Δ in all panels represents GFAPγR1Δ mice treated with isotype control mAb. (TIF 153 kb)

## Abbreviations

BR: Brain
EAE: Experimental autoimmune encephalomyelitis
EDNR_B_: Endothelin-1 receptor B
ET-1: Endothelin-1
GFAP: Glial fibrillary acidic protein
IL: Interleukin
MS: Multiple sclerosis
OLG: Oligodendrocytes
OPC: Oligodendrocyte progenitor cell
P-MS: Progressive multiple sclerosis
SC: Spinal cord
TNF: Tumor necrosis factor
TNFR1: TNF receptor 1
TNFR2: TNF receptor 2

## References

[1] Mahad DH, Trapp BD, Lassmann H (2015). Pathological mechanisms in progressive multiple sclerosis. Lancet Neurol.

[2] Lassmann H, van Horssen J, Mahad D (2012). Progressive multiple sclerosis: pathology and pathogenesis. Nat Rev Neurol.

[3] Plantone D (2016). Secondary progressive multiple sclerosis: definition and measurement. CNS Drugs.

[4] Kutzelnigg A, Lassmann H (2014). Pathology of multiple sclerosis and related inflammatory demyelinating diseases. Handb Clin Neurol.

[5] Lassmann H (2010). Axonal and neuronal pathology in multiple sclerosis: what have we learnt from animal models. Exp Neurol.

[6] Lassmann H (2013). Pathology and disease mechanisms in different stages of multiple sclerosis. J Neurol Sci.

[7] Thompson AJ (2017). Challenge of progressive multiple sclerosis therapy. Curr Opin Neurol.

[8] Boyd A, Zhang H, Williams A (2013). Insufficient OPC migration into demyelinated lesions is a cause of poor remyelination in MS and mouse models. Acta Neuropathol.

[9] Correale J, Farez MF (2015). The role of astrocytes in multiple sclerosis progression. Front Neurol.

[10] El Waly B (2014). Oligodendrogenesis in the normal and pathological central nervous system. Front Neurosci.

[11] Lampron A (2015). Inefficient clearance of myelin debris by microglia impairs remyelinating processes. J Exp Med.

[12] Munzel EJ, Williams A (2013). Promoting remyelination in multiple sclerosis-recent advances. Drugs.

[13] Ozawa K (1994). Patterns of oligodendroglia pathology in multiple sclerosis. Brain.

[14] Larochelle C (2016). Secondary progression in multiple sclerosis: neuronal exhaustion or distinct pathology?. Trends Neurosci.

[15] Rangachari M, Kuchroo VK (2013). Using EAE to better understand principles of immune function and autoimmune pathology. J Autoimmun.

[16] Simmons SB (2013). Modeling the heterogeneity of multiple sclerosis in animals. Trends Immunol.

[17] Holtmann MH, Neurath MF (2004). Differential TNF-signaling in chronic inflammatory disorders. Curr Mol Med.

[18] Miller NM (2015). Anti-inflammatory mechanisms of IFN-gamma studied in experimental autoimmune encephalomyelitis reveal neutrophils as a potential target in multiple sclerosis. Front Neurosci.

[19] Lundgaard I (2014). White matter astrocytes in health and disease. Neuroscience.

[20] Probert L (2015). TNF and its receptors in the CNS: the essential, the desirable and the deleterious effects. Neuroscience.

[21] Ludwin SK (2016). Astrocytes in multiple sclerosis. Mult Scler.

[22] Lassmann H (1999). The pathology of multiple sclerosis and its evolution. Philos Trans R Soc Lond Ser B Biol Sci.

[23] Kimelberg HK, Nedergaard M (2010). Functions of astrocytes and their potential as therapeutic targets. Neurotherapeutics.

[24] Sofroniew MV, Vinters HV (2010). Astrocytes: biology and pathology. Acta Neuropathol.

[25] Hamby ME, Sofroniew MV (2010). Reactive astrocytes as therapeutic targets for CNS disorders. Neurotherapeutics.

[26] Hindinger C (2012). IFN-gamma signaling to astrocytes protects from autoimmune mediated neurological disability. PLoS One.

[27] Savarin C (2015). Astrocyte response to IFN-gamma limits IL-6-mediated microglia activation and progressive autoimmune encephalomyelitis. J Neuroinflammation.

[28] Valentin-Torres A (2016). Sustained TNF production by central nervous system infiltrating macrophages promotes progressive autoimmune encephalomyelitis. J Neuroinflammation.

[29] Selmaj K (1991). Identification of lymphotoxin and tumor necrosis factor in multiple sclerosis lesions. J Clin Invest.

[30] Spuler S (1996). Multiple sclerosis: prospective analysis of TNF-alpha and 55 kDa TNF receptor in CSF and serum in correlation with clinical and MRI activity. J Neuroimmunol.

[31] Begum F (2013). Elevation of tumor necrosis factor alpha in dorsal root ganglia and spinal cord is associated with neuroimmune modulation of pain in an animal model of multiple sclerosis. J NeuroImmune Pharmacol.

[32] Probert L (1995). Spontaneous inflammatory demyelinating disease in transgenic mice showing central nervous system-specific expression of tumor necrosis factor alpha. Proc Natl Acad Sci U S A.

[33] Akassoglou K (1997). Astrocyte-specific but not neuron-specific transmembrane TNF triggers inflammation and degeneration in the central nervous system of transgenic mice. J Immunol.

[34] Dal Canto RA (1999). Local delivery of TNF by retrovirus-transduced T lymphocytes exacerbates experimental autoimmune encephalomyelitis. Clin Immunol.

[35] Selmaj KW, Raine CS (1988). Tumor necrosis factor mediates myelin and oligodendrocyte damage in vitro. Ann Neurol.

[36] Selmaj K, Raine CS (1988). Tumor necrosis factor mediates myelin damage in organotypic cultures of nervous tissue. Ann N Y Acad Sci.

[37] Hisahara S (1997). ICE/CED-3 family executes oligodendrocyte apoptosis by tumor necrosis factor. J Neurochem.

[38] Akassoglou K (1998). Oligodendrocyte apoptosis and primary demyelination induced by local TNF/p55TNF receptor signaling in the central nervous system of transgenic mice: models for multiple sclerosis with primary oligodendrogliopathy. Am J Pathol.

[39] Korn T, Magnus T, Jung S (2005). Autoantigen specific T cells inhibit glutamate uptake in astrocytes by decreasing expression of astrocytic glutamate transporter GLAST: a mechanism mediated by tumor necrosis factor-alpha. FASEB J.

[40] Bonora M (2014). Tumor necrosis factor-alpha impairs oligodendroglial differentiation through a mitochondria-dependent process. Cell Death Differ.

[41] Kim S (2011). Astrocytes promote TNF-mediated toxicity to oligodendrocyte precursors. J Neurochem.

[42] Nakazawa T (2006). Tumor necrosis factor-alpha mediates oligodendrocyte death and delayed retinal ganglion cell loss in a mouse model of glaucoma. J Neurosci.

[43] TNF neutralization in MS: results of a randomized, placebo-controlled multicenter study. The Lenercept Multiple Sclerosis Study Group and The University of British Columbia MS/MRI Analysis Group*.* Neurology, 1999. 53(3):457–465. 10449104

[44] Arnett HA (2001). TNF alpha promotes proliferation of oligodendrocyte progenitors and remyelination. Nat Neurosci.

[45] Gao H (2017). Opposing functions of microglial and macrophagic TNFR2 in the pathogenesis of experimental autoimmune encephalomyelitis. Cell Rep.

[46] Brambilla R (2011). Inhibition of soluble tumour necrosis factor is therapeutic in experimental autoimmune encephalomyelitis and promotes axon preservation and remyelination. Brain.

[47] Karamita M, Barnum C, Möbius W, et al. Therapeutic inhibition of soluble brain TNF promotes remyelination by increasing myelin phagocytosis by microglia. JCI Insight. 2017;2(8):e87455. 10.1172/jci.insight.87455 PMC5396518 28422748

[48] Alexopoulou L (2006). Transmembrane TNF protects mutant mice against intracellular bacterial infections, chronic inflammation and autoimmunity. Eur J Immunol.

[49] Kassiotis G, Kollias G (2001). Uncoupling the proinflammatory from the immunosuppressive properties of tumor necrosis factor (TNF) at the p55 TNF receptor level: implications for pathogenesis and therapy of autoimmune demyelination. J Exp Med.

[50] Chen X (2013). TNFR2 is critical for the stabilization of the CD4+Foxp3+ regulatory T. Cell phenotype in the inflammatory environment. J Immunol.

[51] Chopra M (2016). Exogenous TNFR2 activation protects from acute GvHD via host T reg cell expansion. J Exp Med.

[52] Eugster HP (1999). Severity of symptoms and demyelination in MOG-induced EAE depends on TNFR1. Eur J Immunol.

[53] Liu J (1998). TNF is a potent anti-inflammatory cytokine in autoimmune-mediated demyelination. Nat Med.

[54] D'Haeseleer M (2013). Cerebral hypoperfusion in multiple sclerosis is reversible and mediated by endothelin-1. Proc Natl Acad Sci U S A.

[55] Hammond TR (2014). Astrocyte-derived endothelin-1 inhibits remyelination through notch activation. Neuron.

[56] Hindinger C (2005). Astrocyte expression of a dominant-negative interferon-gamma receptor. J Neurosci Res.

[57] Payne SC (2013). Early proliferation does not prevent the loss of oligodendrocyte progenitor cells during the chronic phase of secondary degeneration in a CNS white matter tract. PLoS One.

[58] Dombrowski Y (2017). Regulatory T cells promote myelin regeneration in the central nervous system. Nat Neurosci.

[59] Gonzalez JM (2005). Expression of a dominant negative IFN-gamma receptor on mouse oligodendrocytes. Glia.

[60] Kapil P (2012). Oligodendroglia are limited in type I interferon induction and responsiveness in vivo. Glia.

[61] Lull ME, Block ML (2010). Microglial activation and chronic neurodegeneration. Neurotherapeutics.

[62] Schmied M (1993). Apoptosis of T lymphocytes in experimental autoimmune encephalomyelitis. Evidence for programmed cell death as a mechanism to control inflammation in the brain. Am J Pathol.

[63] Bauer J (1998). T-cell apoptosis in inflammatory brain lesions: destruction of T cells does not depend on antigen recognition. Am J Pathol.

[64] Bauer J (1999). Apoptosis of T lymphocytes in acute disseminated encephalomyelitis. Acta Neuropathol.

[65] Madsen PM (2016). Oligodendroglial TNFR2 mediates membrane TNF-dependent repair in experimental autoimmune encephalomyelitis by promoting oligodendrocyte differentiation and remyelination. J Neurosci.

[66] Hostenbach S (2016). The pathophysiological role of astrocytic endothelin-1. Prog Neurobiol.

[67] Guo Y (2014). Endothelin-1 overexpression exacerbate experimental allergic encephalomyelitis. J Neuroimmunol.

[68] Shin T (2001). Intrathecal administration of endothelin-1 receptor antagonist ameliorates autoimmune encephalomyelitis in Lewis rats. Neuroreport.

[69] Pache M (2003). Extraocular blood flow and endothelin-1 plasma levels in patients with multiple sclerosis. Eur Neurol.

[70] Haufschild T (2001). Increased endothelin-1 plasma levels in patients with multiple sclerosis. J Neuroophthalmol.

[71] Marsden PA, Brenner BM (1992). Transcriptional regulation of the endothelin-1 gene by TNF-alpha. Am J Phys.

[72] Virdis A (2015). Tumour necrosis factor-alpha participates on the endothelin-1/nitric oxide imbalance in small arteries from obese patients: role of perivascular adipose tissue. Eur Heart J.

[73] Soldano S (2016). Alternatively activated (M2) macrophage phenotype is inducible by endothelin-1 in cultured human macrophages. PLoS One.

[74] Hammond TR (2015). Endothelin-B receptor activation in astrocytes regulates the rate of oligodendrocyte regeneration during remyelination. Cell Rep.

[75] van Oosten BW (1996). Increased MRI activity and immune activation in two multiple sclerosis patients treated with the monoclonal anti-tumor necrosis factor antibody cA2. Neurology.

[76] Walker JE, Giri SN, Margolin SB (2005). A double-blind, randomized, controlled study of oral pirfenidone for treatment of secondary progressive multiple sclerosis. Mult Scler.

[77] Walker JE, Margolin SB (2001). Pirfenidone for chronic progressive multiple sclerosis. Mult Scler.

[78] Batoulis H (2014). Blockade of tumour necrosis factor-alpha in experimental autoimmune encephalomyelitis reveals differential effects on the antigen-specific immune response and central nervous system histopathology. Clin Exp Immunol.

[79] Luo B (2004). ET-1 and TNF-alpha in HPS: analysis in prehepatic portal hypertension and biliary and nonbiliary cirrhosis in rats. Am J Physiol Gastrointest Liver Physiol.

[80] Helset E (1993). Endothelin-1 stimulates human monocytes in vitro to release TNF-alpha, IL-1beta and IL-6. Mediat Inflamm.

[81] Shinagawa S (2017). T cells upon activation promote endothelin 1 production in monocytes via IFN-gamma and TNF-alpha. Sci Rep.

[82] Speciale L (2000). Endothelin and nitric oxide levels in cerebrospinal fluid of patients with multiple sclerosis. J Neuro-Oncol.

[83] Rogers SD (1997). Expression of endothelin-B receptors by glia in vivo is increased after CNS injury in rats, rabbits, and humans. Exp Neurol.

[84] Koyama Y (2013). Different actions of endothelin-1 on chemokine production in rat cultured astrocytes: reduction of CX3CL1/fractalkine and an increase in CCL2/MCP-1 and CXCL1/CINC-1. J Neuroinflammation.

[85] Luscher TF, Barton M (2000). Endothelins and endothelin receptor antagonists: therapeutic considerations for a novel class of cardiovascular drugs. Circulation.

[86] Rodriguez-Pascual F (2004). Transforming growth factor-beta induces endothelin-1 expression through activation of the Smad signaling pathway. J Cardiovasc Pharmacol.

[87] Neumann H, Kotter MR, Franklin RJ (2009). Debris clearance by microglia: an essential link between degeneration and regeneration. Brain.

[88] Puntambekar SS, Hinton DR, Yin X, et al. Interleukin‐10 is a critical regulator of white matter lesion containment following viral induced demyelination. Glia. 2015;63(11):2106–20. 10.1002/glia.22880 PMC4755156 26132901

[89] Shin DI (1999). Interleukin 10 inhibits TNF-alpha production in human monocytes independently of interleukin 12 and interleukin 1 beta. Immunol Investig.

