# Supplementary figures and images for: Blockade of sustained tumor necrosis factor in a transgenic model of progressive autoimmune encephalomyelitis limits oligodendrocyte apoptosis and promotes oligodendrocyte maturation
Source: J Neuroinflammation. 2018 Apr 24;15:121. doi: 10.1186/s12974-018-1164-y (PMC5916830; doi:10.1186/s12974-018-1164-y)

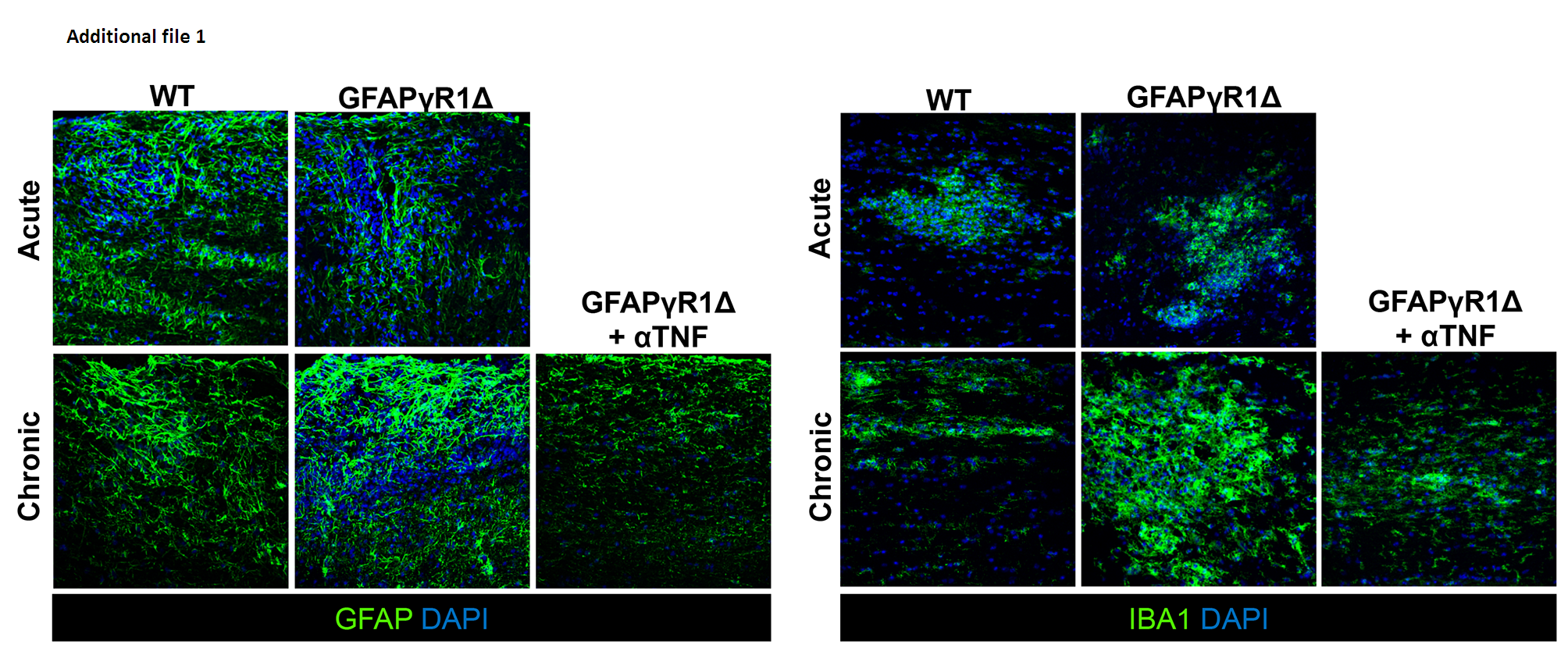

Supplement: Supplementary file 1 — TNF blockade reduces astrocyte and myeloid cell reactivity during progressive EAE in GFAPγR1Δ mice. Longitudinal spinal cord sections from WT and GFAPγR1Δ mice treated with isotype control or anti-TNF mAb were stained for astrocyte (GFAP) or myeloid cell (Iba-1) reactivity at d19 and d30 as indicated. Data represents three to four separate fields per mouse with two to three mice per group. (TIF 1843 kb) [file 12974_2018_1164_MOESM1_ESM.tif]

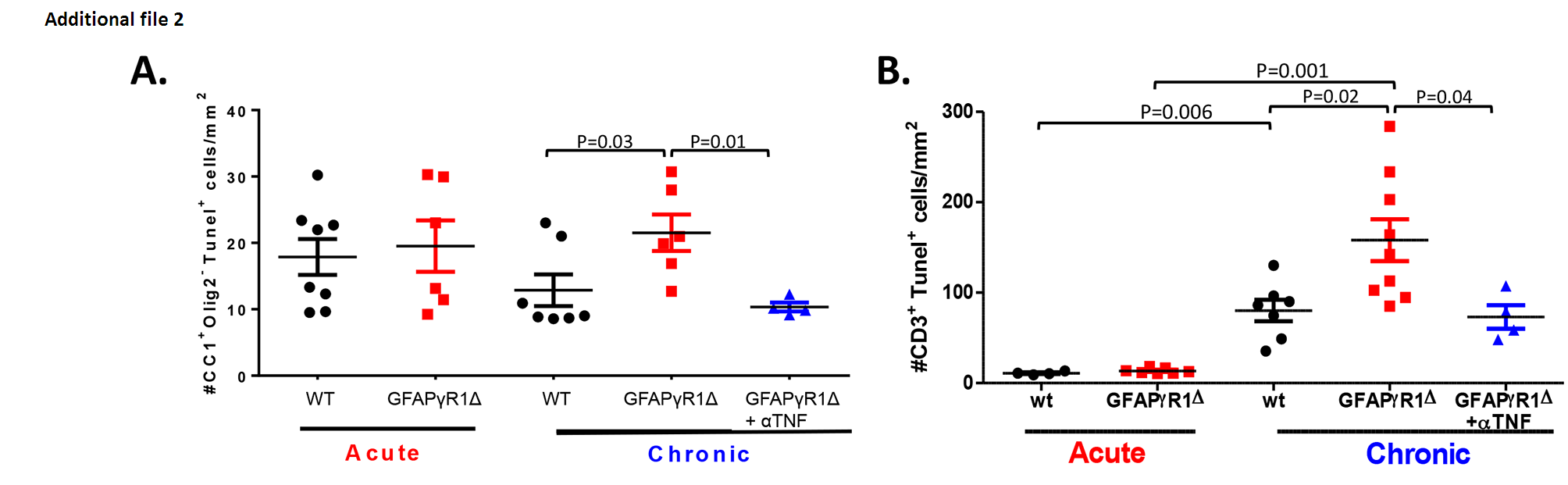

Supplement: Supplementary file 2 — TNF neutralization limits OLG and T cell apoptosis during progressive EAE in GFAPγR1Δ mice. Longitudinal spinal cord sections from WT and GFAPγR1Δ mice, treated with isotype control or anti-TNF mAb, were stained for apoptosis during acute (d19) and chronic (d30) EAE. A. Quantification of apoptotic mature OLG (TUNEL+ CC1+ Olig2−) per square millimeter non-lesioned area. C. Quantification of apoptotic T cells (TUNEL+ CD3+) per square millimeter lesion area. Data represent mean ± SEM of two to three separate fields per mouse with two to three mice per group from two independent experiments. P values were determined by Student’s t test. GFAPγR1Δ in all panels represents GFAPγR1Δ mice treated with isotype control mAb. (TIF 133 kb) [file 12974_2018_1164_MOESM2_ESM.tif]

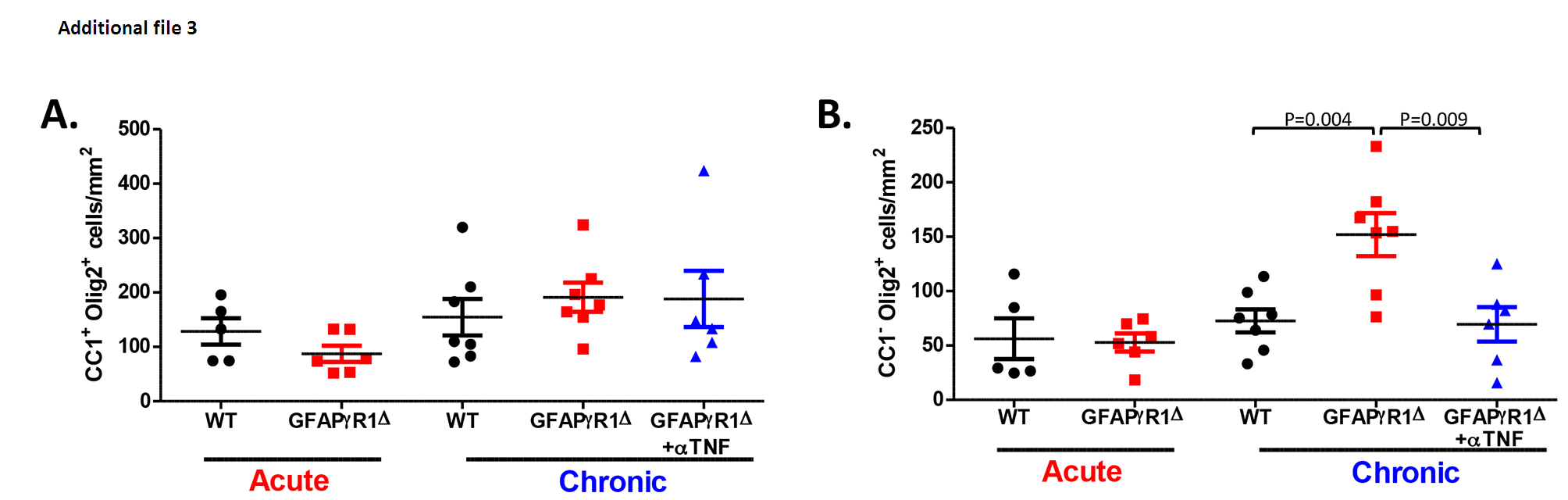

Supplement: Supplementary file 3 — OPC maturation and recruitment is not impaired in non-lesioned areas during progressive EAE in GFAPγR1Δ mice. Longitudinal spinal cord sections from WT and GFAPγR1Δ mice, treated with isotype control or anti-TNF mAb, were stained for OLG and OPC during acute (d19) and chronic (d30) EAE. A. Quantification of differentiated myelinating OLG (CC1+Olig2+) per square millimeter non-lesioned area. D. Quantification of OPCs (CC1−Olig2+) per square millimeter non-lesioned area. Data represent the mean ± SEM of five to seven separate fields per mouse with two to three mice per group from two independent experiments. P values were determined by Wilcoxon rank sum-test. GFAPγR1Δ in all panels represents GFAPγR1Δ mice treated with isotype control mAb. (TIF 153 kb) [file 12974_2018_1164_MOESM3_ESM.tif]
